# New Dimensions in Diabetic Kidney Disease Research: Advanced Organoids and Organs-on-a-Chip

**DOI:** 10.34133/bmef.0296

**Published:** 2026-08-31

**Authors:** Suyan Duan, Mengchu Tang, Ningning Wang, Tingyu Xu, Mengyun He, Shihui Xu, Juan Zhang, Zhongze Gu, Zaozao Chen, Yun Liu

**Affiliations:** ^1^ Department of Nephrology, First Affiliated Hospital with Nanjing Medical University (Jiangsu Province Hospital), Nanjing 210029, China.; ^2^ Division of Nephrology, Department of Geriatrics, First Affiliated Hospital with Nanjing Medical University (Jiangsu Province Hospital), Nanjing 210029, China.; ^3^ Department of Information, First Affiliated Hospital with Nanjing Medical University (Jiangsu Province Hospital), Nanjing 210029, China.; ^4^ State Key Laboratory of Digital Medical Engineering, School of Biological Science and Medical Engineering, Southeast University, 210096 Nanjing, Jiangsu, China.; ^5^ Jiangsu Avatarget Biotechnology Co., Ltd., Suzhou, China.; ^6^ Key Laboratory of Environmental Medicine Engineering of Ministry of Education, School of Public Health, Southeast University, 210096 Nanjing, Jiangsu, China.

## Abstract

Diabetic kidney disease has increasingly emerged as a global public health concern, yet substantial challenges persist in its mechanistic research and drug development. Traditional 2-dimensional cell cultures and animal models frequently lack the capacity to faithfully recapitulate human pathophysiological conditions, and the use of animal models is increasingly limited by ethical and policy-related hurdles. In vitro models such as organoids and organs-on-a-chip have demonstrated remarkable advantages, enabling more faithful simulation of the in vivo microenvironment. The advancement of 3-dimensional bioprinting, microfluidic, and vascularization technologies has further propelled the maturation and progress of in vitro kidney models. This review summarizes and discusses in vitro kidney models and their advances, including kidney cell lines, spheroids, kidney organoids, and kidneys-on-a-chip. It also elaborates on the establishment of diabetic kidney disease models based on these in vitro platforms, which provides robust support for both basic and clinical research.

## Introduction

Due to the worldwide prevalence of diabetes, diabetic kidney disease (DKD), as one of its major complications, has become a major cause of chronic kidney disease and end-stage kidney disease [1]. DKD is associated with poor quality of life, substantial economic burden, and a heightened risk of premature death, making it a globally recognized public health concern [2]. Although intensive glycemic control and blood pressure management are cornerstone strategies for DKD prevention and treatment, they often fail to completely arrest disease progression. Therefore, there is an urgent need for more effective, pathogenesis-targeted therapies. The emergence of novel glucose-lowering agents, such as sodium-glucose cotransporter 2 (SGLT2) inhibitors and glucagon-like peptide-1 receptor (GLP-1R) agonists, has revolutionized traditional treatment paradigms and offers substantial opportunities to reduce the risk of kidney disease progression and mortality [3]. Nevertheless, many novel agents have failed to achieve their primary efficacy endpoints in clinical trials and have raised safety concerns [1]. For instance, praliciguat, which markedly attenuated proteinuria in preclinical models, did not demonstrate a substantial reduction in urine albumin–creatinine ratio in clinical trials [4]. Similarly, bardoxolone methyl, a nuclear factor erythroid 2-related factor 2 (Nrf2) activator, was discontinued in a phase 3 study in patients with DKD (AYAME) owing to an unexpected increase in heart failure events [5]. These examples underscore the persistent translational and safety challenges in DKD drug development. Thus, the prevention, management, and reversal of DKD remain challenging, and considerable efforts are still required.

In vitro studies offer the primary advantage of eliminating harm to patients while allowing efficacy and toxicity assessments. However, despite the widespread use of 2-dimensional (2D) cell cultures and animal models, drug attrition rates remain high, largely owing to their limited predictive capacity for human physiology and pathology [6–8]. Two-dimensional human cell-based models lack intercellular cross talk, cell–matrix interactions, and dynamic physicochemical cues, hampering translation to in vivo conditions [9]. Conventional rodent models for DKD, which are typically established through chemical, surgical, genetic, pharmacological, or dietary interventions, fail to fully recapitulate the entire spectrum of pathological features seen in human DKD [10]. Moreover, animal models raise ethical and cost concerns, and regulatory agencies are actively promoting their replacement. Notably, the Food and Drug Administration (FDA) has announced a policy to phase out animal testing in favor of more predictive, human-relevant models for drug development, and the National Institutes of Health has ceased funding for proposals relying solely on animal testing [11,12]. In 2025, the FDA further issued draft guidance recommending organoids and organ chips as substitutes for nonhuman primates in toxicity testing for certain monoclonal antibodies [13]. Addressing these challenges requires the development of more human-relevant in vitro models to reduce reliance on animal testing in experimental and pharmaceutical research.

A new generation of human-relevant kidney models is emerging, exemplified by kidney organoids, spheroids, and kidney-on-a-chip technologies. These models are generally constructed via the directed differentiation of stem cells induced by exogenous factors. The activated pathways drive the self-organization of tissue constructs engineered to recapitulate the native kidney [14]. Microphysiological systems (MPSs) represent the convergence of microsystems engineering, tissue engineering, and stem cell biology. By integrating biological components such as primary cells, cell lines, or organoids with microfluidic technology, MPSs overcome the limitations of 2D models and enable long-term, stable maintenance of tissue and organ functions. These advanced capabilities allow for more accurate recapitulation of human pathophysiology and improved prediction of therapeutic responses [15]. Microfluidic and organ-on-a-chip (OoC) technologies have shown considerable promise for disease modeling, drug screening, and personalized medicine [16].

**Fig. 1.**
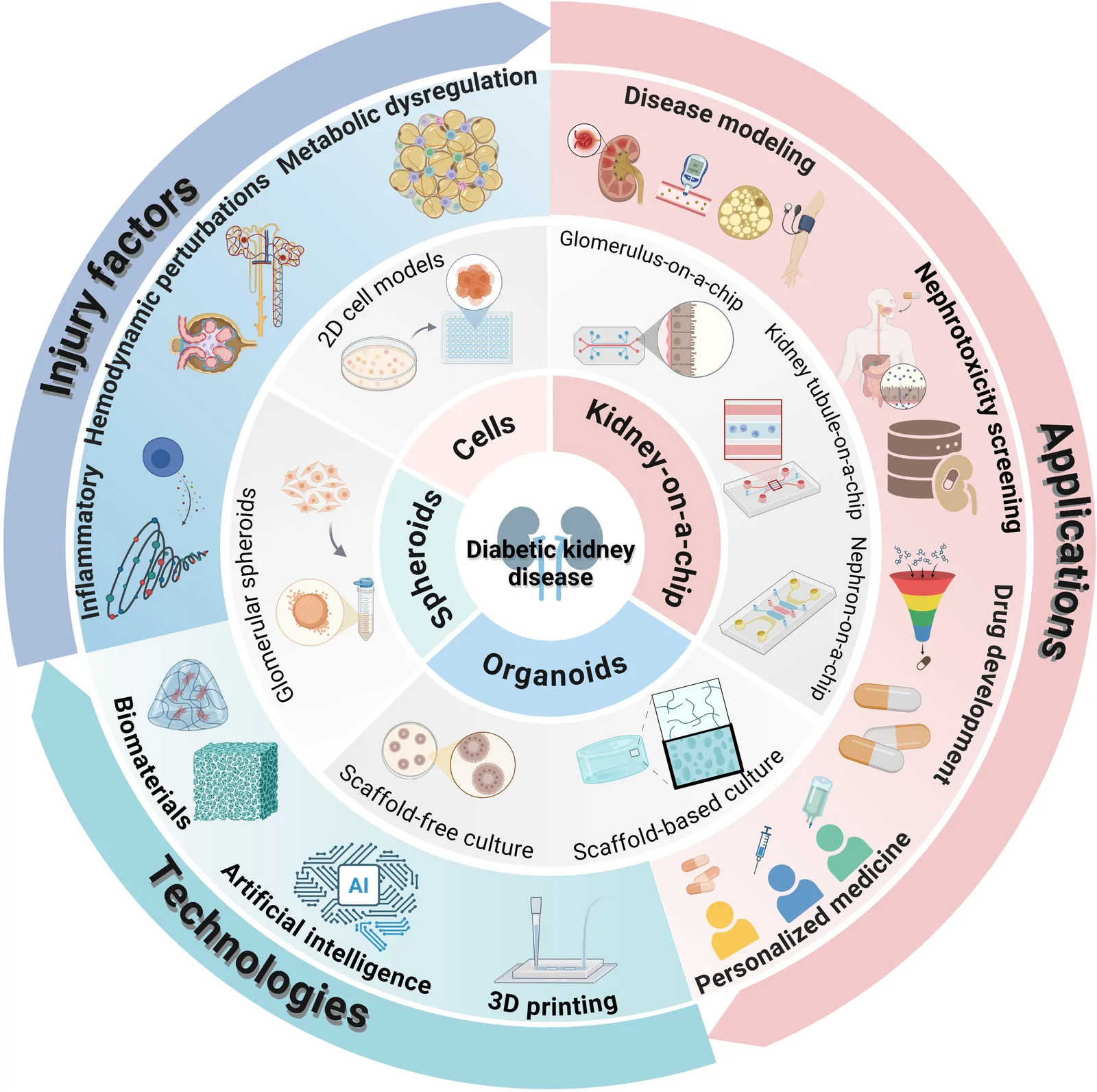
A schematic summary of in vitro model construction and application in diabetic kidney disease (DKD). Common injury factors of DKD include metabolic disorders, hemodynamic disturbances, and inflammatory responses. The commonly used model systems encompass traditional 2-dimensional (2D) cell culture models, kidney spheroids, organoids, and kidneys-on-a-chip, which are widely applied in disease modeling, drug screening, and personalized medicine. Created with BioRender.com, with permission.

Therefore, this review provides a critical and comparative evaluation of major in vitro kidney models, ranging from conventional 2D cultures to spheroids, organoids, and OoC systems, with a specific emphasis on their application in DKD. We systematically assess each model’s capacity to recapitulate DKD-relevant pathological hallmarks and analyze their respective strengths and limitations for mechanistic studies and drug screening. These physiologically relevant platforms facilitate the dissection of DKD pathogenesis, drug candidate evaluation, and toxicity testing, thereby accelerating the development of targeted therapies for improved patient outcomes (Fig. 1).

## Conventional 2D Kidney Cell Culture Models

The widespread use of 2D kidney cell culture models facilitates in-depth mechanistic studies of kidney disease and efficient drug screening. Conventional 2D cultures are typically established using primary cells, cell lines, or stem cells. The selection of cell types is primarily based on their critical roles in the pathophysiological processes of kidney diseases, including glomerular mesangial cells, endothelial cells, podocytes, and tubular epithelial cells (Table 1) [17,18]. Primary cells isolated directly from renal tissue retain native physiological properties, making them valuable for pathophysiological studies [19]. Using primary mouse podocytes, Xu et al. [20] found that high glucose (HG) up-regulates the podocyte-specific transmembrane protein Golgi membrane protein 1 (GOLM1), which exacerbates inflammation, oxidative injury, and apoptosis, highlighting the critical role of podocyte injury in DKD. Similarly, Pan et al. [21] established an in vitro model of DKD with primary human proximal tubule epithelial cells stimulated by HG and transforming growth factor-β1 (TGF-β1) and demonstrated that SGLT2 inhibition regulates fibrosis. However, the short lifespan and limited proliferative capacity of primary cells hinder long-term mechanistic studies and reproducible drug validation experiments. Continuous cell lines, with their unlimited proliferative potential, provide stable and scalable alternatives for mechanistic exploration and drug screening [22]. For instance, Liu et al. [23] showed that the herbal compound wogonin alleviates HG-induced inflammation, apoptosis, and autophagy in mouse podocyte MPC5 cells via B-cell lymphoma-2 (Bcl-2)-dependent signaling. Likewise, Pei et al. [24] uncovered the functional role of a specific protease and its underlying molecular mechanisms in DKD via HG-treated HK-2 cell line models, offering a promising therapeutic target for DKD. Furthermore, human induced pluripotent stem cells (hiPSCs) overcome the proliferative limits of primary cells and the nonphysiological nature of conventional lines, offering unlimited renewal, patient specificity, and superior predictive power for toxicity and therapeutics [25]. This potential has been recently validated in a study where hiPSC-derived podocytes were exposed to multiple injury stressors [26]. The study identified neuraminidase 1 (NEU1) as a conserved target of diabetic podocyte injury, with confirmation in patient kidney biopsies, underscoring the translational value of hiPSC-based models for DKD.

**Table 1.** Construction of 2D and 3D in vitro kidney models

Dimension	Cell category	Cell subtype	Objective	Model	Cell source	Assay methods	Year	Ref.
2D	Primary cells	Primary podocytes	Disease mechanism/pathophysiological research	Cell model	Mouse primary podocytes	Immunofluorescence; cell viability and cell damage assay; intracellular ROS assay; Western blot; RT-PCR; RNA-seq	2025	[20]
		Primary glomerular endothelial cells	Disease mechanism/pathophysiological research	Cell model	Human primary glomerular endothelial cells (hPGECs)	RT-PCR; ELISA; Western blot	2022	[123]
		Proximal tubular epithelial cells (PTECs)	Drug development/renal toxicity evaluation	Cell model	Human PTECs	Cell viability and LDH leakage assay; RT-PCR; endocytic receptor function assay; flux measurement	2020	[124]
	Cell lines	Podocyte cell line	Mechanism-based drug action research	Cell model	Mouse podocyte clone-5 (MPC5)	Transmission electron microscopy; cell viability assay; Western blot; RT-PCR; immunofluorescence; flow cytometry	2022	[23]
			Mechanism-based drug action research	Cell model	Human podocytes (AB8/13)	Immunofluorescence; cell migration assay; ELISA; Western blot; apoptosis and cell cycle assay	2020	[125]
			Mechanism-based drug action research	Cell model	Immortalized human podocyte cells (HPCs)	Immunofluorescence; Western blot; cell viability analysis; intracellular ROS assay	2024	[126]
		Glomerular endothelial cell line	Mechanism/pathophysiological research	Cell model	Immortalized mouse glomerular endothelial cells (mGECs)	RT-PCR	2023	[127]
		PTEC line	Mechanism/pathophysiological research	Cell model	Human renal PTECs (HK-2)	Western blot; RT-PCR; immunofluorescence	2021	[128]
			Drug development/renal toxicity evaluation	Cell model	Porcine renal PTECs (LLC-PK1)	Cell viability assay; intracellular ROS assay; Western blot	2020	[129]
	Pluripotent stem cells (PSCs)	Human induced pluripotent stem cells (hiPSCs)	Disease modeling	Cell model	hiPSC-derived podocytes	Immunocytochemistry; flow cytometry; Western blot; albumin uptake assay; RT-PCR; cell viability assay; cytotoxicity assay	2022	[19]
		hiPSCs	Renal toxicity evaluation	Cell model	hiPSC-derived renal PTECs	Cell viability assay; RT-PCR; immunofluorescence; Western blot; flow cytometry; albumin uptake assay	2023	[130]
3D	Primary cells	Primary glomerular endothelial cells; primary PTECs; primary podocytes	Renal toxicity evaluation	Microphysiological systems	Primary podocytes, vascular endothelial cells, and tubular epithelial cell from male SD rats	Immunocytochemistry; cell viability assay; cytotoxicity assay	2018	[83]
	Cell lines	Podocyte cell line; PTEC line	Design and development of renal microphysiological systems	Microphysiological systems	Conditionally immortalized human podocytes (CIHP-1) and human proximal tubule cells (HK-2)	Cell viability and proliferation assay; fluorescent staining	2021	[131]
		PTEC line	Design and development of renal organoids	Organoid	Immortalized human renal PTECs (RPTEC-TERT1)	Cell damage assay; genetic analysis; phenotypic high-content imaging; protein assay	2022	[132]
	Stem cells	hiPSCs	Design and development of renal organoids	Organoid	hiPSCs: WTC11 and CMC11	Immunofluorescent analysis; RT-PCR; Western blot; transmission electron microscopy; single-cell RNA-seq	2022	[133]
		hiPSCs	Design and development of organ-on-a-chip	Organ-on-a-chip	hiPSC line: PGP-1	Immunofluorescence; ELISA; scanning electron microscopy; transmission electron microscopy	2024	[68]
		Human mesenchymal stem cells (hMSC)	Design and development of renal spheroids	Spheroid	hMSCs (Lonza)	Immunofluorescence; RT-PCR; cell viability assay	2019	[33]

2D, 2-dimensional; 3D, 3-dimensional; ROS, reactive oxygen species; RT-PCR, reverse transcription polymerase chain reaction; RNA-seq, RNA sequencing; ELISA, enzyme-linked immunosorbent assay; LDH, lactate dehydrogenase; SD, Sprague Dawley

Conventional 2D monolayer cultures remain indispensable in early-stage mechanistic research and high-throughput drug screening, owing to their simple operation, cost-effectiveness, and rapid establishment [27]. Nevertheless, these conventional systems still have several limitations. First, primary-cell-derived 2D models exhibit considerable inter-individual variability, which leads to interbatch discrepancies in cell differentiation and biomarker expression [28]. Immortalized cell lines and hiPSC-derived cells are homogeneous. However, they lack the multisystem interactions, intercellular communication, and mechanical signals present in vivo, thus failing to recapitulate the complex pathophysiological regulatory networks [27,29]. This deficiency in architectural and functional complexity largely accounts for the high failure rate of translating preclinical findings from 2D systems to clinical applications. Therefore, there is a pressing need to develop more physiologically relevant in vitro models that better recapitulate the native tissue microenvironment.

## Spheroids and Organoids

Compared with 2D models, 3-dimensional (3D) models can more accurately simulate tissue development, disease phenotypes, and the delivery and penetration of drug compounds in vivo (Table 1). The key advantage of 3D models lies in their capacity to mimic and modulate multicellular cross talk and physiological microenvironments, which enables them to highly recapitulate human physiological and pathological states [30]. In recent years, advances in microfluidics and kidney cell biology have propelled the development of sophisticated dynamic models. Consequently, kidney spheroids, organoids, and kidney-on-a-chip systems have been widely adopted in nephrology, contributing to the refinement and reduction of animal experiments [31].

### Spheroids

Spheroids are self-assembled, spherical cell aggregates that provide inherent advantages of 3D culture through their ability to model the architecture and microenvironment of native tissues [32]. Glomerular spheroids are 3D cell culture systems composed of kidney-derived cells. They can be formed through cell self-assembly regulated by natural extracellular matrix (ECM) signals in suspension or directed assembly assisted by biomaterials [33]. Such models can stably maintain a spherical microstructure, simulate the kidney in physical form, and recapitulate some of its functions. Kang et al. [34] generated functional glomerular spheroids from kidney-derived cell lines in an engineered ECM under shear stress. Using this procedure, spheres formed within 24 h and remained stable for at least 5 d. This platform mimics the renal 3D structure and native ECM, overcoming 2D limitations and better recapitulating kidney function. Giannuzzi et al. [35] isolated renal progenitor cells from the urine of healthy controls and patients with kidney disease. These cells were shown to be capable of intrinsic spheroid formation without exogenous chemokine stimulation. Furthermore, they express markers typical of pluripotent cells, such as stage-specific embryonic antigen 4 (SSEA4), secrete elevated levels of renin, and exhibit angiogenic properties. Notably, renal progenitor cells isolated from the urine of patients with immunoglobulin A (IgA) nephropathy form spheroids capable of recapitulating the characteristic IgA1 deposition observed in this disease, thereby suggesting the potential of spheroid models for studying the mechanisms of kidney diseases and for drug discovery and also providing insights for DKD research. By utilizing HG media to mimic the hyperglycemic microenvironment of DKD, researchers have identified a key protease closely implicated in renal fibrosis and inflammation in human kidney tubular spheroids, thereby supporting the development of targeted protease inhibitors for DKD therapy [36]. Such spheroid models are simple to establish and highly cost-effective, and they also exhibit higher cell viability and model stability compared to 2D culture methods. Additionally, researchers have developed a rapidly generated and highly reproducible 3D co-culture spheroid model [37]. Through artificial induction or co-culture with plasma from patients with diverse disease phenotypes, this model is suitable for rapid pharmacological screening for DKD-related therapeutic agents, representing a promising, high-throughput-compatible platform for drug screening and disease modeling. Despite these promising applications, the adoption of spheroid models in kidney research lags considerably behind their widespread use in oncology [38]. This disparity may stem largely from the inherent architectural and functional complexity of the kidney, particularly the glomerular filtration barrier (GFB) and the intricate tubular–vascular interplay [18]. Such complexity may not be adequately recapitulated by the relatively simple spherical aggregates of a single or few cell types. Moreover, DKD modeling demands sustained exposure to metabolic stressors over days to weeks to elicit clinically relevant fibrotic and inflammatory phenotypes [24]. The limited long-term culture stability and functional maturation of spheroids severely restrict their utility in chronic disease modeling and long-term drug efficacy evaluation.

### Kidney organoids

Organoids are organ-like structures spontaneously formed in vitro based on 3D cell culture methods using embryonic stem cells or induced pluripotent stem cells (iPSCs) [39]. Kidney organoids comprise multiple renal cell types and recapitulate the complex tissue architecture and physiological functions of the human kidney.

**Fig. 2.**
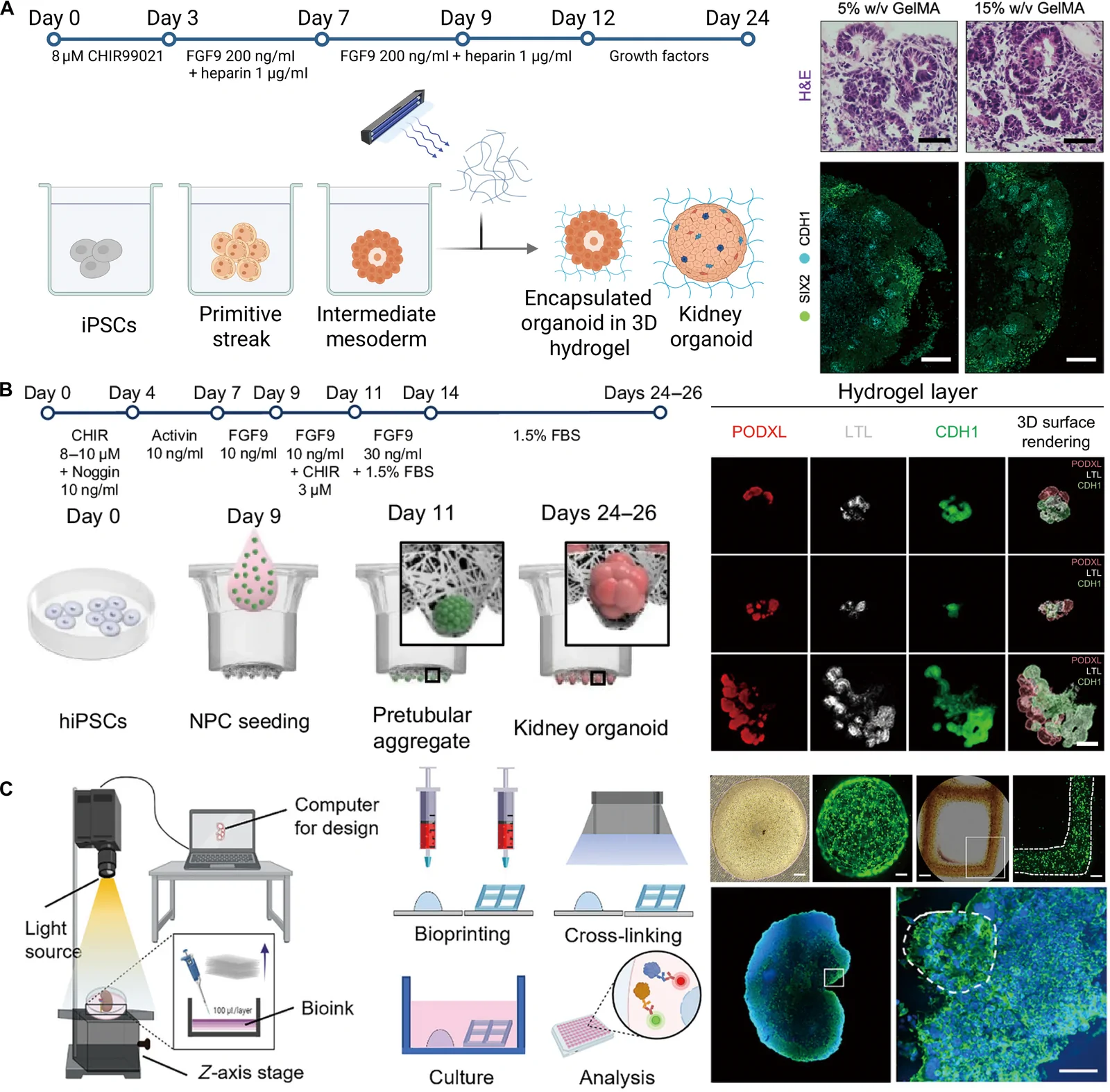
Methods of kidney organoid culture. (A) The scaffold-based culture method promotes the growth and differentiation of kidney organoids. The images on the right show hematoxylin and eosin (H&E) staining (scale bar: 50 μm) and immunofluorescence staining (scale bar: 150 μm) of the self-organization level of kidney organoids after scaffold-based culture. Reproduced with permission [44]. Copyright 2025, *Biomaterials*. (B) Scaffold-free embedded culture method. The 3-dimensional (3D) surface-rendered confocal imaging on the right demonstrates that kidney organoids cultured based on microwell arrays exhibit a distinct cellular structural distribution. Scale bar: 100 µm. Reproduced with permission [53]. Copyright 2024, *Nature Communications*. (C) Extrusion-based bioprinting process. The bright-field and fluorescence images on the right display cell growth and distribution after bioprinting, and confocal imaging reveals 3D multilayered miniature renal microtissues fabricated via bioprinting. Scale bars: 100 µm. Reproduced with permission [56]. Copyright 2025, *Advanced Healthcare Materials*. Created with BioRender.com, with permission.

In vitro 3D cell culture methods generally include scaffold-based culture and scaffold-free culture [38]. The natural ECM comprises a complex mixture of structural proteins, growth factors, and proteoglycans, which collectively form a dynamic, pericellular structure that delivers tissue-specific microenvironmental cues [40]. Decellularized ECM (dECM) is a biomaterial obtained by removing immunogenic cellular components through decellularization technology [41]. By mimicking the native microenvironment of kidney development, naturally derived or synthetic ECM provides 3D support for in vitro kidney organoid culture [42]. Garreta et al. [43] harvested dECM from porcine and human kidneys. Histological and immunohistochemical analyses verified that decellularized renal scaffolds preserved basement membrane proteins consistent with native renal tissue. With dECM hydrogel supplementation, the team efficiently generated human-pluripotent-stem-cell-derived kidney organoids containing podocyte-like and tubular-like cells. Immunofluorescence validated the expression of canonical renal markers, including podocyte markers (NEPHRIN, Wilms tumor 1 [WT1], and podocalyxin-like [PODXL]), the proximal tubular marker *Lotus tetragonolobus* lectin (LTL), and distal tubular E-cadherin (ECAD). Clerkin et al. [44] further developed a semisynthetic methacrylated gelatin hydrogel as a novel support matrix (Fig. 2A). This matrix is more stable than conventional dECM materials and provides a tunable mechanical microenvironment. They found that a higher stiffness mimicking that of adult kidneys (5,000 to 10,000 Pa) was more favorable for nephrogenesis and organoid maturation. In contrast, another study used microbial transglutaminase to cross-link gelatin into a stiffness-tunable hydrogel [45]. The study reported that podocytes cultured on substrates mimicking a healthy glomerular basement membrane (2,000 to 5,000 Pa) exhibited up-regulated gene and protein expression. These divergent stiffness optima reported across studies highlight the current lack of standardized biophysical culture parameters for in vitro model systems. Using single-cell RNA sequencing (scRNA-seq), Treacy et al. [46] compared the cellular compositions of organoids cultured in synthetic hydrogels, at the air–liquid interface (Transwell), or in Matrigel. The results indicated that organoids derived from both hydrogels (PeptiGel Alpha 4 and Alpha 5) exhibited high expression of *PITX2* and *SHISA2*, genes linked to nephron progenitor induction and self-renewal. Furthermore, those derived from the Alpha 5 hydrogel with high stiffness showed up-regulated expression of mature podocyte markers, including nephrosis 2 (NPHS2), annexin A1 (ANXA1), and PODXL. These findings indicate that renal cells are highly sensitive to subtle alterations in substrate mechanical properties and that the physicochemical characteristics of the biomaterial per se profoundly influence tissue growth and morphogenesis, ultimately contributing to low organoid viability, developmental immaturity, and substantial batch-to-batch variability [47–49].

Another method is scaffold-free embedded culture, which foregoes exogenous matrices and facilitates self-assembly autonomously through endogenous ECM deposition or via directed physicochemical means [50]. Microwell arrays represent a straightforward and widely adopted implementation approach. Tran et al. [51] constructed a simple, scalable, and cost-effective microwell platform using laser-micromachined EZSPHERE 12-well plates, which can generate more than 5,000 organoids per plate. Nevertheless, constrained by intrinsic dimensional and permeability limitations, conventional microwell architectures fail to sufficiently deliver nutrients, growth factors, and oxygen throughout the constructs. Bioreactors overcome this drawback by enabling bulk organoid production using custom-engineered devices. Specifically, spinner flask bioreactors improve the perfusion of oxygen and nutrients inside renal organoids, permitting parallel generation of hundreds of nephron-structured organoids within a 14-d culture period [52]. Kim et al. [53] developed UniMat, an organoid culture platform featuring a core 3D geometrically engineered permeable membrane (Fig. 2B). The permeable microwell array adopts an acute-angled, narrow-based geometry. This design directs cells toward the center of each well, enhancing cell–cell contact and promoting aggregate formation. Meanwhile, the permeable membrane structure ensures effective diffusion and exchange of nutrients, growth factors, and oxygen, supporting the differentiation and maturation of organoids. UniMat allows medium sharing across microwells. This feature limits the high-content screening of individual organoids. Moreover, mimicking native organ complexity requires more than soluble factors. It demands tissue-specific microenvironments, mechanical cues, and cell–cell interactions. Thus, long-term stability and functional maturation remain major challenges. Addressing these challenges will require integrating dynamic perfusion, mechanical stimulation, and multicellular co-culture strategies into next-generation platform designs.

Microfluidic 3D bioprinting is an emerging biofabrication technology that enables precise spatial orchestration of cells, hydrogels, and bioactive molecules [54]. The standard bioprinting workflow is initiated by imaging the target anatomy, followed by computer-aided design of biomimetic templates, preparation of functional bioinks, and, finally, the printing and in vitro maturation of the organ constructs [55]. Bioink refers to a natural or synthetic material that incorporates cells, bioactive factors, or dECM components. Shin et al. [56] engineered a kidney-derived dECM bioink (kdECMMA) that is bioactive and viscoelastic with a tunable stiffness between 0.67 and 4.81 kPa, ensuring high viability and proliferation in primary human kidney cells (Fig. 2C). Another promising material is alginate, a natural scaffold that has been successfully employed for cultivating a variety of organoids. Hydrogels formed via thiol-ene cross-linking of norbornene-functionalized alginate exhibit a wider tunability in mechanical properties and greater formulation flexibility [57]. By adjusting the dosage of dithiol cross-linkers, the storage modulus of the hydrogel can be precisely regulated from 0.1 to 8 kPa. Formica et al. [58] employed core–shell printing technology to encapsulate a composite metanephric mesenchyme/ureteric bud progenitor cell bioink within an alginate shell and combined it with a microfluidic bioprinter to successfully fabricate cell-laden hollow fibers for the in vitro culture of kidney organoids. The cells in the bioprinted filaments began aggregating within 8.5 h and maintained high viability at 24 h. By day 6, they had progressed to form kidney vesicles and, by day 20, ultimately differentiated into kidney organoids that were morphologically similar to nonbioprinted in vitro cultures.

Despite advances in kidney organoid generation, current models face inherent limitations that impede clinical translation. A major challenge is inadequate vascularization [59]. Although numerous studies have reported vascularized organoids, functional perfusable vascular networks remain lacking, which restricts nutrient and oxygen supply to the organoid interior during extended culture [60]. Moreover, the static ECM, while initially supporting organoid growth, may eventually hinder the establishment of perfusable vascular networks rather than facilitating them [59]. In addition, the absence of immune cells compromises the functional maturation and viability of kidney organoids, thereby limiting their utility in modeling inflammation-related diseases and evaluating immunotherapies [61]. Studies have successfully developed human leukocyte antigen class I negative iPSC-derived kidney organoids for kidney transplantation by knocking out the *B2M* gene using clustered regularly interspaced short palindromic repeats (CRISPR)–Cas9 but failed to completely avoid T-cell-mediated immune rejection, which may be related to the cytotoxic activity of T cells within the tissue [62].

## OoCs and MPSs

An OoC is a microengineered biomimetic system combining stem cell culture with microfluidics, enabling dynamic cell culture via controlled perfusion to model multicellular structures, tissue–tissue interfaces, physicochemical microenvironments, and vascular perfusion [63]. In recent years, various microfluidic OoC models have been developed to mimic the glomerulus, the proximal tubule, the distal tubule, the collecting duct, and even entire nephrons, which provides crucial support for kidney disease modeling and drug nephrotoxicity assessment [64].

### Glomerulus-on-a-chip

**Fig. 3.**
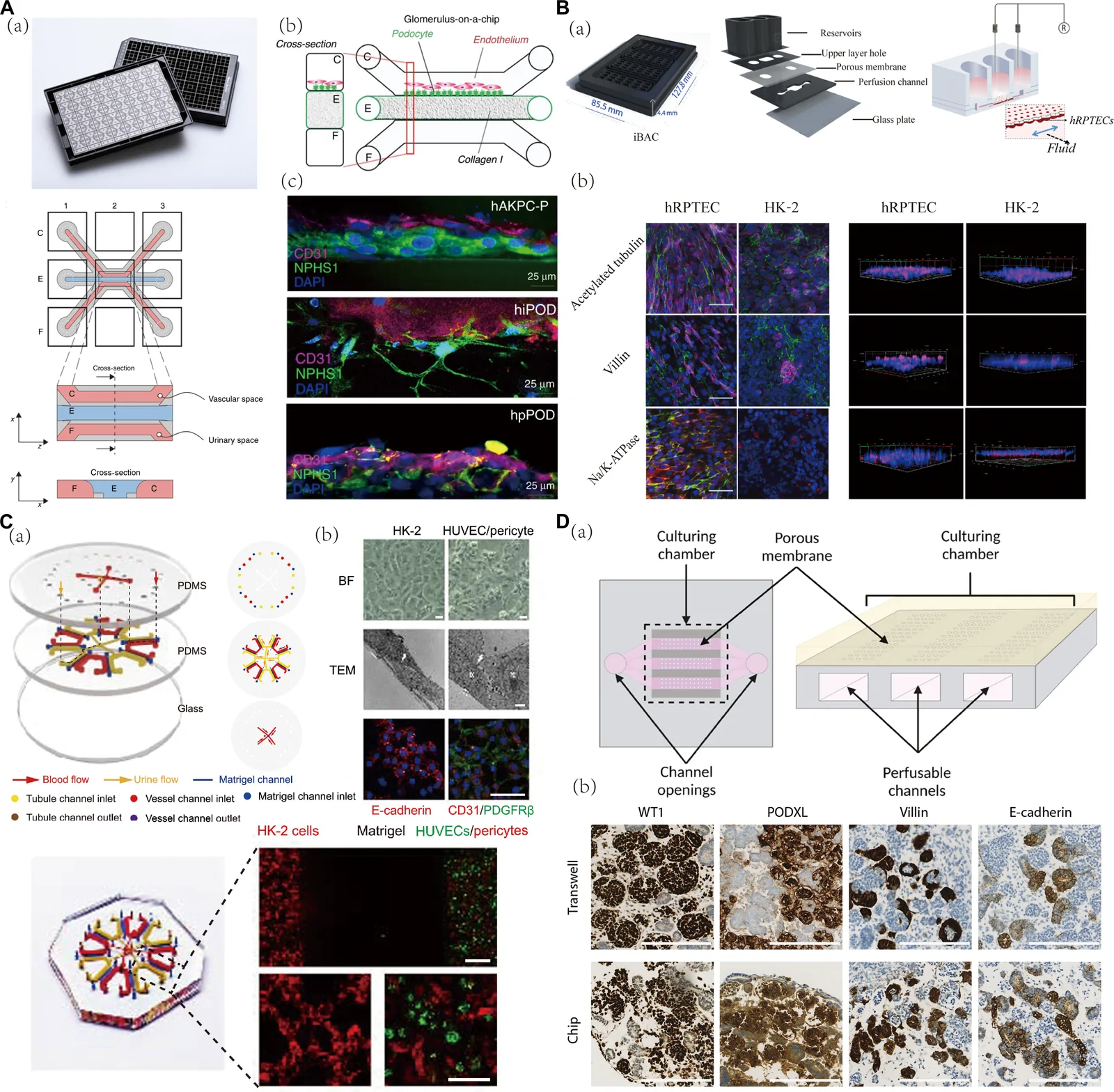
Construction of kidney-on-a-chip models. (A) Glomerulus-on-a-chip with a glomerular filtration barrier. (a) Schematic of chip architecture; (b) podocyte and glomerular endothelial cell seeding; (c) confocal images of cell-specific markers. Reproduced with permission [67]. Copyright 2019, *Nature Communications*. (B) High-throughput human renal proximal tubule chip based on an integrated biomimetic array chip (iBAC). (a) Schematic and exploded view of the chip; (b) immunofluorescence micrographs of human renal proximal tubule epithelial cells (hRPTECs) and HK-2 cells after 4 d of culture. Scale bars = 100 µm. Reproduced with permission [72]. Copyright 2022, *Biosensors* (Basel). (C) Renal tubule–interstitium chip. (a) Schematic of multichannel co-culture configuration showing HK-2 cells, human umbilical vein endothelial cells (HUVECs), and pericytes in separate channels (scale bars: 250 µm [top] and 75 µm [bottom]); (b) immunofluorescence confirming cell growth (scale bars: 75 µm [top]; 2 µm [middle]; 75 µm [bottom]). Reproduced with permission [79]. Copyright 2022, *Acta Biomaterialia*. (D) Kidney organoid vascularization on organ-on-a-chip. (a) Schematic of chip configuration; (b) immunohistochemical staining of kidney organoids showing glomeruli, podocytes, and renal tubules. Scale bars: 200 µm. Reproduced with permission [85]. Copyright 2022, *Scientific Reports*.

The glomerulus is the key research focus in DKD development and progression [65]. The GFB is composed of podocytes, endothelial cells, and the glomerular basement membrane. Damage or disruption of any component will impair the integrity of the GFB, leading to abnormal glomerular filtration rate and elevated creatinine levels [66]. Petrosyan et al. [67] created a functional glomerulus-on-a-chip model by integrating human podocytes and glomerular endothelial cells in the MIMETAS platform (Fig. 3A). This device maintained high cell viability under perfusion for at least 4 weeks and demonstrated differential clearance of albumin and inulin. The self-assembled matrix, composed of collagen IV and laminin, recapitulates the GBM. HG exposure induced barrier injury, and the model further demonstrates its applicability in DKD modeling and GBF-targeted drug screening. Although OoC technology is playing an increasingly important role in kidney development and disease modeling, glomerular-only structures may be incompletely differentiated and are constrained by the absence of mesangial cells and vascular components. Their utility in drug screening remains uncertain. How to ensure the differentiation integrity of these models and accurately recapitulate the native kidney microenvironment remains a critical challenge to be addressed.

Mou et al. [68] fabricated an ultrathin biomimetic basement membrane from silk fibroin to interface the glomerular epithelium with the vascular endothelium in a dynamic microfluidic system. This glomerulus-on-a-chip system successfully recapitulated the tricompartmental structure and filtration function of the GFB. Compared with conventional polydimethylsiloxane (PDMS) membranes, the ultrathin electrospun silk fibroin membrane can be sectioned for electron microscopy, enabling clear observation of endothelial development and intercellular interactions. In a similar manner, researchers have engineered an ex vivo GFB using electrospun biomimetic membranes seeded with human glomerular cells. Novel biomaterials are thinner and more bioactive, making them more attractive than conventional PDMS and polycarbonate membranes. This device coupled with a bioreactor generates bilateral low-velocity flow fields that recapitulate physiological shear stresses—blood flow on endothelial cells and urinary flow on podocytes—thereby promoting the maturation and differentiation of both cell types [69].

By exposing the chips to patient-derived serum, researchers can assess serum-induced nephrotoxicity and associated GFB permeability alterations [70]. This approach recapitulates glomerular pathology and serves as a screening platform for evaluating candidate drug efficacy. Artificial membranes have become increasingly biomimetic in composition, while microfluidic chips now incorporate physiologically relevant shear stresses and mechanical strain. However, most glomerular chips lack essential cellular constituents, including mesangial cells, and key microenvironmental components, such as immune cells [67,70]. This compositional deficiency prevents current models from fully recapitulating the in vivo complexity of the GFB. Therefore, incorporating these missing elements to reconstitute the full glomerular architecture and function is a critical priority for next-generation platform development.

### Kidney tubule-on-a-chip

Kidney tubular injury is increasingly recognized as a driver of kidney disease progression, and the majority of kidney-protective drugs target renal tubules [71]. Based on this, renal tubule chips have shown great potential in kidney disease modeling and drug-induced kidney injury assessment. Jing et al. [72] designed a renal proximal tubule model using a bidirectional rocking-driven integrated biomimetic array chip (iBAC) (Fig. 3B). Compared to models on static Transwell systems, the primary human renal proximal tubule epithelial cells (RPTECs) cultured on the iBAC exhibited a denser morphology and higher cell viability. It also showed high expression of membrane transporters such as Megalin, organic cation transporter 2 (OCT2), and organic anion transporter 1 (OAT1), which better mimic the substance transport scenarios in vivo renal proximal tubules. To further integrate the vascular system into MPSs, researchers fabricated a 3D tubular structure using a collagen hydrogel, enabling the co-culture of renal tubular epithelial cells and vascular endothelial cells under whole-blood perfusion [73]. This human renal vascular–tubular chip combines several features, such as flow, biological ECM components, endothelial cells, and perivascular cells, and can also be used to study renal tubular physiological functions via glucose perfusion. Beyond these capabilities, the vascularized tubule chip enables the tubular secretion of solutes, offering a more comprehensive model for studying drug exposure in the proximal tubule [74]. This integrated platform thus holds promise for applications in kidney disease modeling, pharmaceutical screening, and precision medicine.

Biomedical materials and 3D bioprinting platforms have opened new avenues for manufacturing vascularized organ-specific tissues for both in vitro and eventual in vivo applications [75]. Lin et al. [76] used 3D printing to co-localize vascular and proximal renal tubule channels, encapsulating them in engineered ECM and then using a closed-loop perfusion system to study renal reabsorption. By adjusting the ratio of gelatin and fibrin in the engineered ECM, the epithelial confluence time in this model was reduced by more than 75% compared to that of the native ECM model. Similarly, by co-culturing RPTECs and human umbilical vein endothelial cells (HUVECs) in microchannels within 3D-printed PDMS scaffolds, high cell adhesion was observed on the channel surface within 30 min, with rapid confluence of the cells occurring within 12 h [77]. This device effectively reduced elevated glucose levels, exhibited robust albumin reabsorption, and recapitulated amphotericin B-induced tubular injury. It employs multiple functionalization strategies, such as plasma treatment, aminopropyltriethoxysilane, and type I collagen. These surface modifications enhance cell adhesion, promote proliferation, and support long-term stable attachment, thereby increasing the device’s operational reliability and experimental utility. However, factors such as perfusion volume, microchannel diameters, biomaterial solute absorption, and other variables present challenges to the fidelity of the current model. In addition, interdevice variability, fabrication complexity, and assembly failure rates introduce further uncertainties, affecting reproducibility and cross-study comparability [78]. Therefore, additional engineering advances and refinement of culture conditions are necessary to bridge the gap between chip-based observations and in vivo renal physiology.

Although vascularized chips replicate the symbiotic physiological environment of blood vessels and renal tubules, it remains challenging to fully simulate the complex structure and function inherent to the renal interstitial microenvironment. Liu et al. [79] designed a multilayer PDMS microfluidic chip containing a 3D channel network and 8 simulated renal interstitial tubular–vascular units (Fig. 3C). HK-2 cells were cultured in channels representing renal tubules, while HUVECs and pericytes were cultured in channels representing the vascular system surrounding the renal tubules. Confocal microscopy enabled direct observation of fluorescein isothiocyanate–dextran diffusion across the matrix gel to peritubular capillaries, confirming the passive diffusion process in the renal interstitium. Despite modeling only tubular structure, this chip features low variability, simple fabrication, and scalability and has been used to study the proteinuria–tubular injury–peritubular microvascular injury cascade. To minimize the impact of nonrenal cell types (such as HUVECs) on model fidelity, Bouwens et al. [80] developed a chip that enables co-culture of multiple human kidney-derived cell types. The proximal tubular epithelial cells, renal endothelial cells, and platelet-derived growth factor receptor beta (PDGFRβ)-positive perivascular mesenchymal cells derived from kidney biopsies were immortalized and co-cultured in 3D-bioprinted constructs, demonstrating the key driving mechanisms of tubular–interstitial interactions and renal fibrosis. Notably, immune cells are equally critical in driving fibrosis but were not included in this study. This is also a common limitation shared by many current models. Beyond cellular composition, the 3D microenvironment itself must also be optimized, as it can respond more sensitively to nephrotoxic stimuli, placing higher demands on the composition and structure of the ECM. Adjusting the properties of biomaterials and identifying optimal parameters will help construct a microenvironment that is more susceptible to pathological alterations [81].

### Nephron-on-a-chip

The nephron is the basic functional unit of the kidney, consisting of the glomerulus, Bowman’s capsule, proximal convoluted tubule (PCT), Henle’s loop, and distal convoluted tubule [82]. Constructing a complete in vitro nephron model with intact structure and function to achieve complex filtration, reabsorption, secretion, and collection functions is a highly challenging task. Qu et al. [83] developed a nephron-on-a-chip model using primary cells derived from mice, which includes components of the glomerulus, Bowman’s capsule, the proximal renal tubule lumen, and peritubular capillaries. Through fluorescence imaging, they identified VE-cadherin, E-cadherin, and podocin, confirming tight junctions between the components. Additionally, this chip simulates nephrotoxic drug metabolism via dynamic circulating artificial blood flow and glomerular filtrate, showing superior results compared to those of traditional kidney tubule chips. The human kidney cell line co-culture system integrates glomerular and proximal tubular compartments within a microphysiological platform that replicates physiological flow, shear stress, and pressure. Relative to the mouse primary-cell-based model, this system offers greater translational relevance for human drug screening. Using the MPS, researchers have successfully simulated and maintained key renal physiological parameters, including a PCT shear stress of 0.4 to 1.5 dynes/cm^2^, a flow split of 64.0% flow recirculation and 36.0% filtration output, and a glomerular pressure of 9.66 ± 4.12 mmHg [84]. In this model, the classic nephrotoxic agents cisplatin and doxorubicin were tested. Proteinuria and glycosuria were observed across all drug-treated conditions, indicating that the compounds induced concurrent damage to both the GBM and the PCT. These findings provide an initial proof of concept for this platform in preclinical drug screening.

To further address the limitations of organ chips in cellular heterogeneity, functional integrity, and vascularization, incorporating kidney organoids into microfluidic organ chips for culture is an innovative approach (Fig. 3D) [85]. Kidney organoids derived from iPSCs can be differentiated on the chip and exhibit multiple nephron segments. Co-culture with HUVECs under microfluidic flow regulates endothelial cell infiltration and migration, facilitating the formation of engineered endothelial-derived vascular networks. Moreover, the microfluidic OoC typically employs an automated pump for continuous perfusion culture, eliminating the need for manual medium changes, which substantially reduces hands-on intervention and lowers the risk of contamination [86].

Due to the kidney’s cellular complexity and high vascularization, constructing an in vitro model that fully represents the entire renal cell population is challenging [87]. While organoids or their derived cells have been used in microfluidic chips, the differentiation and development of complex cellular aggregates introduce issues of reproducibility and batch variability [88]. These represent primary hurdles that must be addressed to ensure reliable preclinical application of kidney-on-a-chip platforms.

## In Vitro Modeling Strategies for DKD

The diabetic environment is primarily driven by HG conditions. Combined exposure to HG, TGF-β, advanced glycation end-product-modified albumin, hypoxia, angiotensin II, and other diabetic mediators provides deeper insights into DKD [89]. For instance, HG medium has been shown to inhibit the viability of immortalized mouse podocytes (MPC) in a time-dependent manner [90]. This DKD cell model has further been employed to investigate the anti-inflammatory and anti-apoptotic properties of pharmaceutical compounds that attenuate HG-induced renal cell damage.

**Fig. 4.**
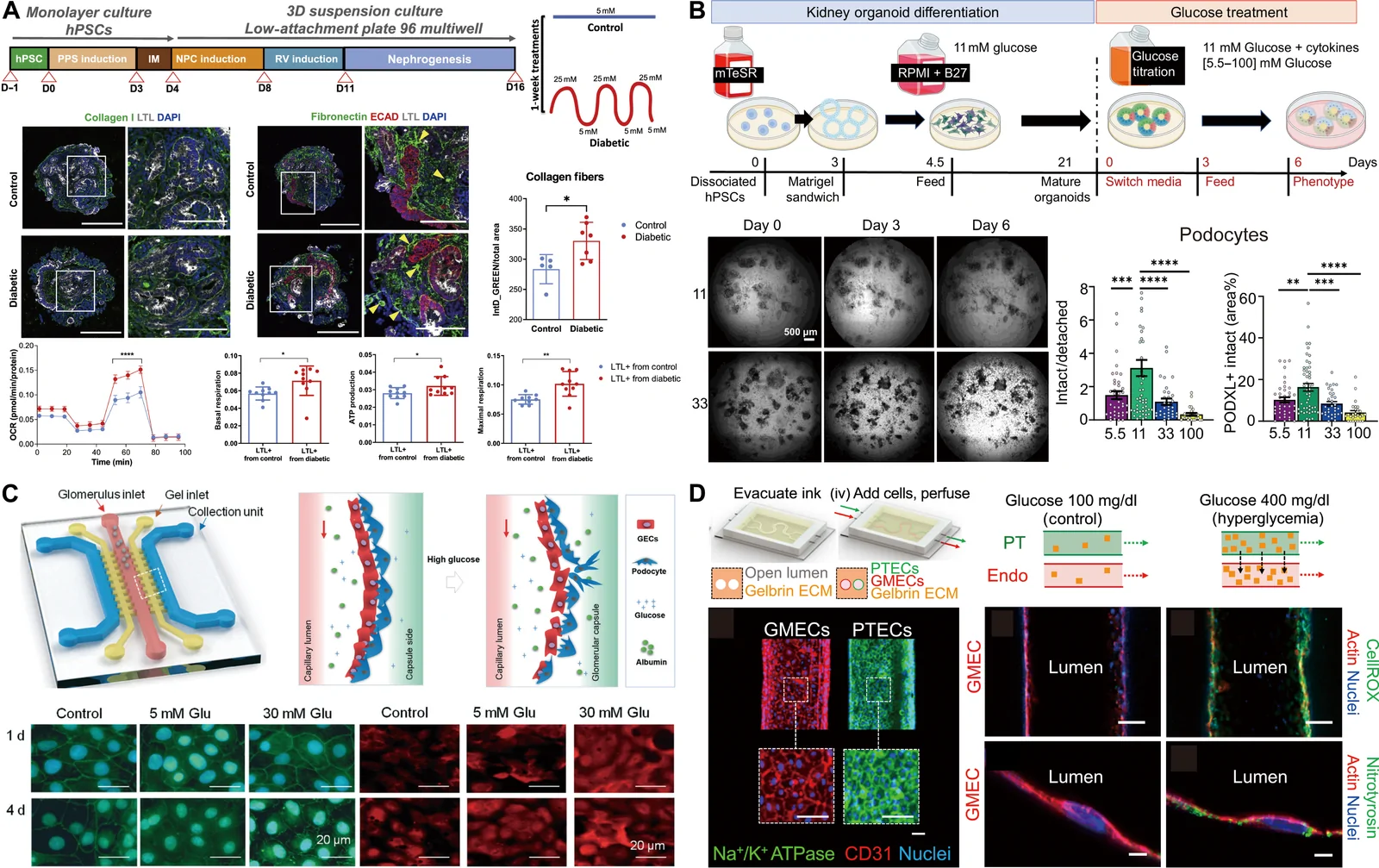
Establishment of diabetic kidney disease (DKD) models in organoids and organs-on-a-chip. (A) Human kidney organoids exposed to highly oscillatory glucose conditions show elevated collagen and fibronectin deposition, accompanied by transcriptional changes, extracellular matrix (ECM) remodeling, and mitochondrial metabolic reprogramming, as shown by immunofluorescence and quantification. Scale bars: 250 and 100 µm. *P < 0.05; **P < 0.005; ****P < 0.0001. Reproduced with permission [91]. Copyright 2022, *Cell Metabolism*. (B) High-glucose (HG)-induced intrinsic inflammation in kidney organoids leads to epithelial degradation and podocyte loss, as demonstrated by time-lapse phase-contrast imaging, recapitulating the early pathophysiological features of diabetic nephropathy. **P < 0.01; ***P < 0.001; ****P < 0.0001. Reproduced with permission [92]. Copyright 2025, bioRxiv. (C) Glomerulus-on-a-chip reconstituting the glomerular filtration barrier (GFB) for early diabetic nephropathy modeling, with HG conditions increasing GFB permeability and reducing the expression of epithelial barrier integrity markers and glucose transport/metabolism markers. Reproduced with permission [95]. Copyright 2017, *Lab on a Chip*. (D) Vascularized renal tubule chip subjected to cyclic HG perfusion induces endothelial cell injury, with immunofluorescence staining revealing oxidative stress in glomerular endothelial cells under hyperglycemic conditions. Scale bars: 100 µm (left); 100 µm (upper right); 1 µm (lower right). Reproduced with permission [76]. Copyright 2019, *Proceedings of the National Academy of Sciences of the United States of America*.

To more accurately simulate the fluctuating blood glucose levels in diabetic patients, Garreta et al. [91] designed a method for culturing kidney organoids under intermittent HG conditions (alternating between 5 and 25 mM every 24 h) (Fig. 4A). This highly oscillating glucose treatment led to increased collagen fiber deposition, reconfiguration of mitochondrial metabolic profiles, and up-regulated angiotensin-converting enzyme 2 (ACE2) expression at both the messenger RNA and protein levels. Collectively, these alterations rendered engineered human kidney organoids phenotypically similar to the progression of DKD.

DKD involves not only hyperglycemia but also multiple mechanistic pathways, including inflammation and epithelial cell dysfunction. Spennati et al. [92] exposed organoid models to pro-inflammatory cytokines (tumor necrosis factor-α [TNF-α], TGF-β, and macrophage migration inhibitory factor [MIF]) across a range of glucose concentrations (Fig. 4B). Notably, these cytokines induced podocyte injury even at physiologically relevant glucose levels, while subsequent scRNA-seq demonstrated that hyperglycemia further up-regulated TNF-α and MIF expression, exacerbating inflammatory responses and podocyte damage. Using this platform, the researchers demonstrated that the TNF-α inhibitor etanercept and the MIF inhibitor ISO-1 exerted protective effects, restoring PODXL+ intact regions under 33 mM HG conditions to levels comparable to those of normal cultures. In another study investigating diabetic vascular injury, organoid models were similarly subjected to HG and cytokine stimulation for the screening of multiple hypoglycemic drugs and small-molecule inhibitors [93]. Numerous glucose-lowering agents were investigated, and the γ-secretase inhibitor DAPT was identified as capable of markedly suppressing abnormal type IV collagen accumulation and rescuing endothelial cell proliferation in diabetic vascular organoids. However, a standardized strategy for the directional perfusion of organoid vasculature and nephron segments is lacking. The inability to recapitulate native nephron fluid dynamics precludes accurate in vitro assessment of drug uptake and transport kinetics, thereby limiting the translational utility of these models for drug screening, disease modeling, and renal repair.

Organ chips provide a high-fidelity, high-throughput innovative platform for DKD modeling and drug research (Table 2) [94]. Using a glomerular chip to model early-stage DKD, Wang et al. [95] employed bovine serum albumin as a permeability tracer and, with real-time high-resolution imaging, found that HG stimulation (30 mM) markedly elevated GFB albumin leakage, accompanied by GFB dysfunction and reactive oxygen species production (Fig. 4C). These changes closely recapitulate the hallmark proteinuria and pathological responses observed in human diabetic nephropathy. Proximal tubules play a crucial role in reabsorption and transport functions, responsible for nearly 90% of glucose reabsorption in the kidney [96]. Lin et al. [76] infused a glucose solution at a concentration of 400 mg/dl into the channels of a vascularized proximal tubule model and observed that microvascular endothelial cells exhibited oxidative stress and disrupted junctions (Fig. 4D). They also found that treatment with dapagliflozin restored endothelial function, providing evidence for targeted therapies for microvascular damage in DKD. Advanced 3D chips exhibit selective reabsorption and solute carrier-mediated transport. Equipped with precisely regulated microfluidic perfusion for controlled solute and drug delivery, these platforms enable the quantitative assessment of intercellular cross talk and drug efficacy under physiological and pathological conditions.

**Table 2.** Applications of organs-on-a-chip in the construction of DKD models

Type	Disease model	Source	Intervention factors	Culture time	Assay methods	Applications	Ref.
Glomerulus-on-a-chip	Diabetic nephropathy model	Rat primary glomeruli	High-glucose media: 5.5 mM (control), 10.5 mM, 35.5 mM	4 and 15 d	Cell viability assay; albumin filtration assay; cell migration assay; immunofluorescence	Elucidate the mechanism of diabetic nephropathy; support drug toxicity testing	[95]
Glomerulus-on-a-chip	Diabetic nephropathy model	Human primary glomerular endothelial cells; human primary podocytes; human immortalized podocyte lines; human amniotic-fluid-derived podocytes	High-glucose media: 10 mM (control), 15 mM, 20 mM	72 h	Albumin filtration assay	Replicate glucose-induced damage	[67]
Renal tubule-interstitium-on-a-chip	Renal tubulointerstitial fibrosis model	Human proximal tubular epithelial cell lines; human kidney endothelial cell lines; human perivascular PDGFRβ renal mesenchymal cell lines	TGF-β stimulation: 10 ng/ml Ochratoxin A stimulation: 10 μM	24 and 16 h	qPCR; immunofluorescence	Model human kidney fibrosis in vitro; develop novel targeted anti-fibrotic therapies	[80]
Vascularized proximal tubule-on-a-chip	Hyperglycemia-induced model	Human immortalized PTECs; human primary GMECs	High-glucose media: 100 mg/dl (control), 400 mg/dl with and without dapagliflozin	15 d	ELISA; confocal microscope-based imaging assay; transmission electron microscopy	Explore the effect of hyperglycemia on cellular function; support drug research	[76]
Vascularized proximal tubule-on-a-chip	Proteinuria model	Human immortalized renal proximal tubule epithelial cells (HK-2 cells); human umbilical vascular endothelial cells (HUVECs)	Medium containing 5% non-inactivated healthy human serum (HHS)	24 and 48 h	Immunofluorescence; confocal microscope; living cell tracker staining; cell apoptosis assay	Elucidate the mechanism of proteinuria-induced RIF; identify potential therapeutic targets	[79]
Immunoactive vascularized proximal tubule-on-a-chip	Renal inflammation model	Human RPTECs; HUVECs; human primary monocytes	Medium containing 5% (v/v) cobra-venom-activated human complement serum (CAS)	96 h	Immunofluorescence; lactate dehydrogenase (LDH) release; cytokine release; cell viability assay	Elucidate renal functionality and inflammatory processes; support drug screening	[118]

DKD, diabetic kidney disease; PDGFRβ, platelet-derived growth factor receptor beta; TGF-β, transforming growth factor-β; qPCR, quantitative polymerase chain reaction; PTECs, proximal tubule epithelial cells; GMECs, glomerular microvascular endothelial cells; ELISA, enzyme-linked immunosorbent assay; RIF, renal interstitial fibrosis; RPTECs, renal proximal tubule epithelial cells

## Applications in Drug Discovery and Precision Medicine

**Fig. 5.**
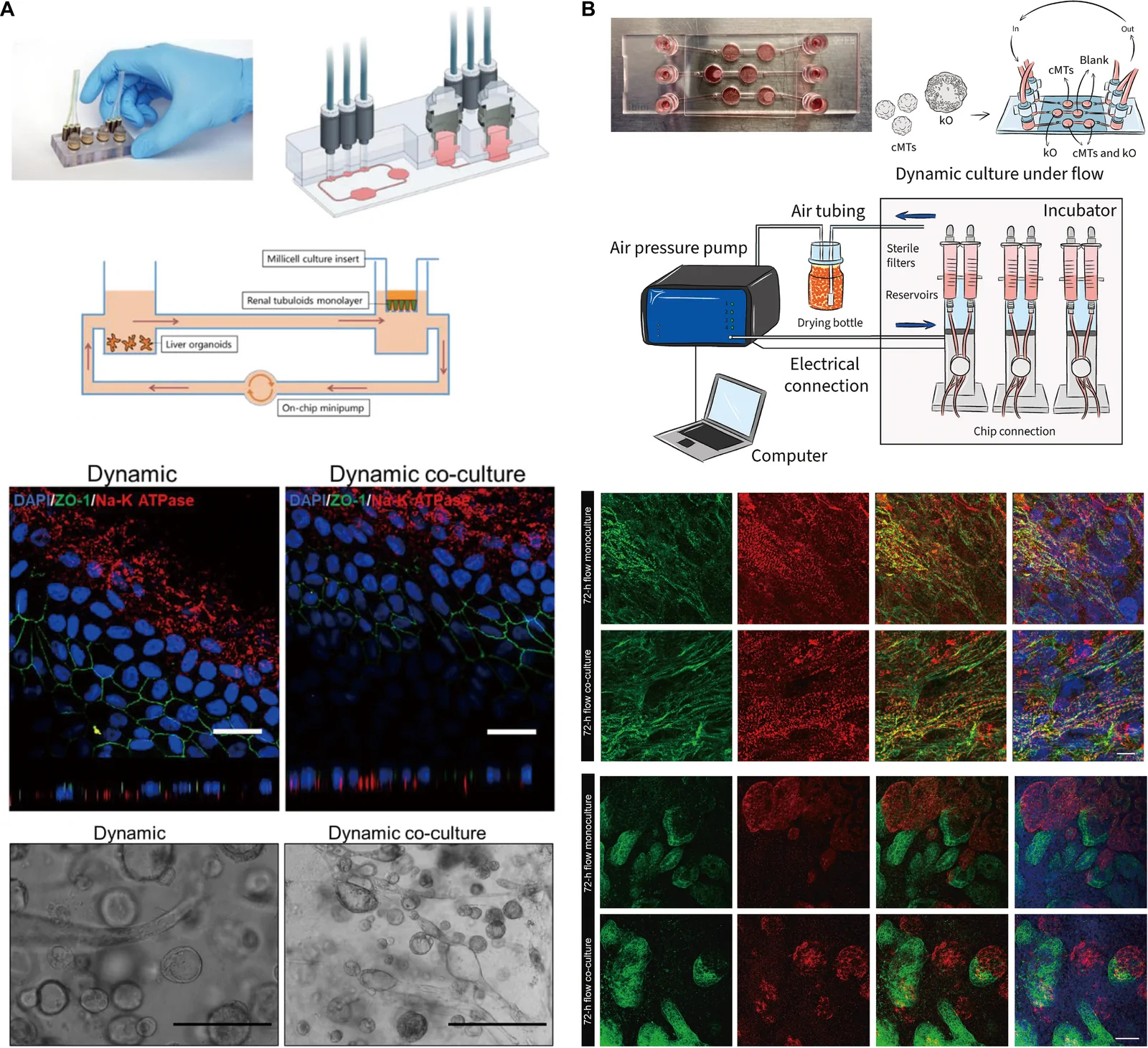
Establishment of multi-organ organ-on-a-chip (OoC) models. (A) A multi-organ chip model based on human kidney and liver organoids. Each chip consists of 2 independent circuits, with each circuit composed of 2 cell culture chambers interconnected via a microchannel system, enabling the co-culture of liver and renal tubule organoids. The lower images show that after dynamic co-culture, the renal tubule organoids differentiated to form an epithelial barrier, while the liver organoids not only remained spherical but also acquired a tubular morphology. Scale bars: 25 µm (top); 1,000 µm (bottom). Reprinted with permission [101]. Copyright 2022, *Journal of Extracellular Vesicles*. (B) Microfluidic chip for a cardio-renal unit. Each chip consists of 3 channels, 2 of which are interconnected to allow for the co-culture of 2 organoids while maintaining their separation. Immunofluorescence staining shown below demonstrates that both the cardiac and renal organoids maintained their respective structural and functional characteristics under both monoculture and co-culture conditions over 72 h. Scale bars: 10 µm (top); 50 µm (bottom). Reprinted with permission [107]. Copyright 2023, *Materials Today Bio*.

OoC models are increasingly being used in drug research, including pharmacokinetics and pharmacodynamics, providing insights for drug screening and new drug development [97]. However, due to the lack of a macroscopic biological context supporting cross talk between different organ systems, single-organ chips still have certain limitations [98]. There are mainly 2 strategies for building multiple organ chips: one is to couple individual organ chip units, and the other is to integrate multiple organ chips into a single culture plate [99]. Chang et al. [100] connected individual liver and kidney chips through polyethylene tubes and used syringe pumps to maintain the perfusion of the chips. This integrated microfluidic system recapitulates organ–organ interactions and elucidates the hepatic bioactivation, interorgan transport, and nephrotoxicity of drugs. Another innovative study developed a single-device integrated liver–kidney-on-a-chip platform, featuring 2 independent microfluidic loops interconnected by a microchannel network [101]. After 10 d of co-culture, the chip successfully supported the differentiation, maturation, and functional establishment of both cell types (Fig. 5A). In contrast, single-device co-cultures generally have smaller volumes and higher throughput and require less medium but are also more difficult to implement [102]. Since the liver and kidney are closely involved in drug metabolism and excretion, liver–kidney chips are widely used for drug toxicity testing [103], especially to assess the renal toxicity effects of liver metabolites [104].

A dynamic multi-organ chip comprising pancreas, adipose tissue, and the liver was developed by 3D cell printing with dECM bioinks [105]. This chip recapitulates type 2 diabetes mellitus (T2DM) under hyperglycemic conditions, incorporates visceral adipose tissue, and includes a separate retinal compartment to monitor diabetic complications. This platform also elucidates the mechanisms of antidiabetic drugs, including gliclazide and metformin. Building on these validated applications, integration of additional relevant organ models would further broaden its utility for studying systemic T2DM complications. Furthermore, multi-organ chip technology has also been used to validate risk factors potentially contributing to diabetes or kidney injury, such as how fine particulate matter (PM2.5) in the air might induce insulin resistance, resulting in metabolic disorders [106]. Similarly, Gabbin et al. [107] developed a microfluidic system to study heart–kidney interactions, which preserved the structural and functional characteristics of both organs under co-culture conditions for investigating the human cardiorenal axis (Fig. 5B). Although most common multi-organ chips typically integrate 2 to 3 organs [108], advanced robotic technologies have enabled the development of a vascularized organ chip incorporating 8 human organs (intestine, liver, kidney, heart, lung, skin, blood–brain barrier, and brain) [109], which provides a more physiologically relevant in vitro platform for systemic drug development. However, multichip linkage may impair organ function. For instance, liver albumin production decreased upon connection to 7 other chips versus a stand-alone chip, indicating that physical interconnection or shared circulation could reduce hepatocyte performance and revealing unresolved issues such as shear stress, medium mismatch, and signal cross talk.

The integration of advanced technologies from various fields with 3D in vitro models provides novel potential strategies for the study of kidney diseases. Visualization techniques commonly used in in vitro model studies include immunofluorescence, high-resolution microscopy, and quantitative polymerase chain reaction [110]. In contrast, scRNA-seq can comprehensively identify the various cell types differentiated in kidney in vitro models, evaluating the cellular composition and gene regulatory networks of kidney organoids [110]. scRNA-seq can also reveal cytokines and signal transduction pathways induced by HG, as well as identify potential therapeutic targets for DKD [111]. Kidney organoid datasets have also been developed, which can be used to improve the cell differentiation protocols in in vitro models [112]. In addition, CRISPR, a highly efficient and targeted gene editing technology [113], has been used to study phenotypes associated with podocyte dysfunction and glomerular diseases in organoids [114]. CRISPR-based functional screens also aid in identifying potential regulatory networks in DKD and exploring the mechanisms by which hyperglycemia exacerbates kidney injury [115].

## Conclusions and Future Perspectives

**Fig. 6.**
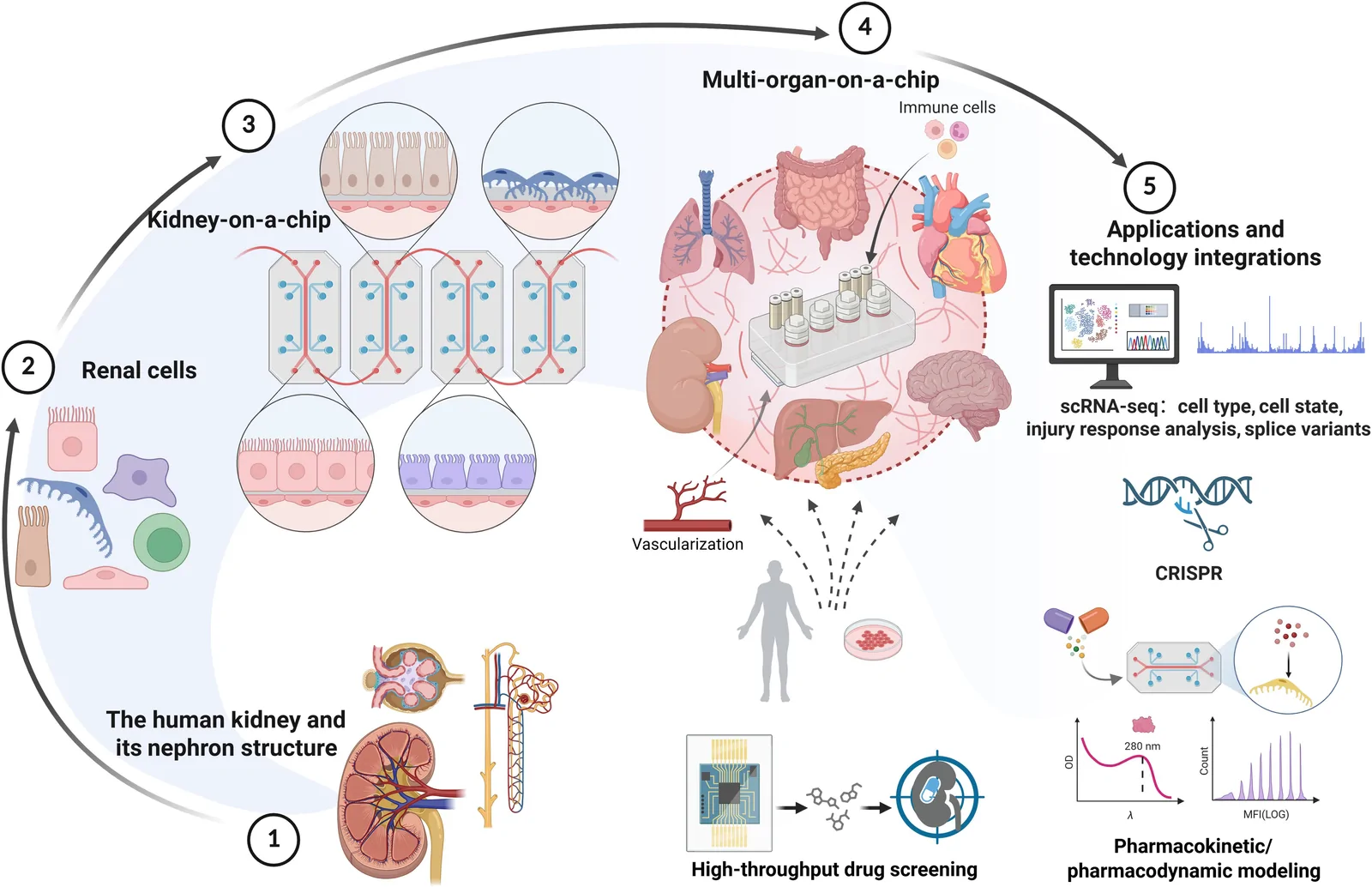
Organ-on-a-chip: construction and future applications. Reproduced with permission [14]. Copyright 2024, *Annual Review of Biomedical Engineering*. Created with BioRender.com, with permission.

In vitro kidney models have brought new dimensions to the study of DKD. The development of kidney organoids and microfluidic kidney chips, along with the integration of technologies such as bioprinting [76], gene editing [115], and artificial intelligence [116,117], has provided strong support for kidney disease modeling, drug screening, and personalized medicine (Fig. 6).

However, these novel kidney in vitro models still face many challenges. The simulation of vascularization and the immune microenvironment, along with their long-term stability, has consistently been a focus of research. Bioprinted tubular structures and co-culture with endothelial cells have partially simulated vascular structures, while microfluidic technology provides a dynamic culture environment for cells by mimicking blood flow [76]. Although existing technologies have somewhat simulated the basic functions of blood vessels, the lack of functional vasculature still leads to insufficient intercellular signaling, limiting the structural maturation and physiological functional homeostasis of in vitro models [14]. Additionally, the immune microenvironment in in vitro models has garnered increasing attention. Co-culture with human primary monocytes can preliminarily assess immune-mediated kidney injury and proximal tubule inflammation [118]. A kidney model co-cultured with T-cell bispecific antibodies (TCBs) and peripheral blood mononuclear cells can be used to evaluate the podocyte-killing effects induced by selective WT1-TCBs [119]. Although the incorporation of immune cells into kidney models has been limited, studies have successfully integrated diverse immune cell types into lung MPSs for investigating infection and inflammation [120]. Therefore, to better replicate the components of the circulatory and immune systems, more realistic microenvironments need to be recreated, aiming to improve the functionality and structural biomimicry of kidney organoids and organ chips.

Moreover, current culture models still have limitations in disease modeling, especially for chronic progressive diseases like DKD. HG perfusion, commonly used to induce DKD models, typically lasts 24 h to 3 weeks [67,92,95], with few reports extending beyond 3 weeks [91], indicating that current models largely capture acute glucose toxicity rather than chronic hyperglycemia. Long-term HG perfusion is therefore expected to become a key future direction [76].

As emerging products, kidney organoids and organ chips need to achieve reliable, economical, scalable, and large-scale manufacturing methods in order to transition from laboratory research to clinical applications [121]. Additionally, challenges such as the source of cells and biomaterials, industrial production standards, and industry regulatory frameworks must be overcome, particularly in the field of drug development [122].

In conclusion, kidney organoids and kidney chips provide innovative tools for DKD research, demonstrating great potential in disease modeling and drug screening. The synergistic effects of various emerging biomedical technologies are collectively driving the translation of advanced 3D in vitro models to clinical applications, ultimately benefiting patients.

## References

[1] Alicic R, Nicholas SB. Diabetic kidney disease back in focus: Management field guide for health care professionals in the 21st century. Mayo Clinic Proc. 2022;97(10):1904–1919. 10.1016/j.mayocp.2022.05.003 36202498

[2] Thomas MC, Brownlee M, Susztak K, Sharma K, Jandeleit-Dahm K, Zoungas S, Rossing P, Groop P-H, Cooper ME. Diabetic kidney disease. Nat Rev Dis Primers. 2015;1(1):15018. 27188921 10.1038/nrdp.2015.18 PMC7724636

[3] Gaddy A, Elrggal M, Madariaga H, Kelly A, Lerma E, Colbert GB. Diabetic kidney disease. Dis Mon. 2025;71(4): Article 101848. 39753456 10.1016/j.disamonth.2024.101848

[4] Xu Y, Wu X, Ge M, Liang K, Xing J, Zhang P, Li H, Guo Z, Mei X. Soluble guanylate cyclase modulators—A novel therapeutic approach for diabetic kidney disease. Ren Fail. 2025;47(1): Article 2595385. 41485909 10.1080/0886022X.2025.2595385 PMC12777800

[5] Guo M, He F, Zhang C. Molecular therapeutics for diabetic kidney disease: An update. Int J Mol Sci. 2024;25(18):10051. 39337537 10.3390/ijms251810051 PMC11431964

[6] Fogel DB. Factors associated with clinical trials that fail and opportunities for improving the likelihood of success: A review. Contemp Clint Trials Commun. 2018;11:156–164. 10.1016/j.conctc.2018.08.001 PMC6092479 30112460

[7] Tagle DA. The NIH microphysiological systems program: Developing *in vitro* tools for safety and efficacy in drug development. Curr Opin Pharmacol. 2019;48:146–154. 31622895 10.1016/j.coph.2019.09.007

[8] Seok J, Warren HS, Cuenca AG, Mindrinos MN, Baker HV, Xu W, Richards DR, McDonald-Smith GP, Gao H, Hennessy L, et al. Genomic responses in mouse models poorly mimic human inflammatory diseases. Proc Natl Acad Sci USA. 2013;110(9):3507–3512. 23401516 10.1073/pnas.1222878110 PMC3587220

[9] Buvall L, Menzies RI, Williams J, Woollard KJ, Kumar C, Granqvist AB, Fritsch M, Feliers D, Reznichenko A, Gianni D, et al. Selecting the right therapeutic target for kidney disease. Front Pharmacol. 2022;13: Article 971065. 36408217 10.3389/fphar.2022.971065 PMC9666364

[10] Noshahr ZS, Salmani H, Rad AK, Sahebkar A. Animal models of diabetes-associated renal injury. J Diabetes Res. 2020;2020:9416419. 32566684 10.1155/2020/9416419 PMC7256713

[11] FDA. FDA announces plan to phase out animal testing requirement for monoclonal antibodies and other drugs. FDA. 10 Apr 2025. [accessed 5 Dec 2025] https://www.fda.gov/news-events/press-announcements/fda-announces-plan-phase-out-animal-testing-requirement-monoclonal-antibodies-and-other-drugs

[12] CN Bio. NIH to prioritize human-based research tech. CN Bio. 7 Jun 2025. [accessed 5 Dec 2025] https://cn-bio.com/nih-to-prioratize-human-based-research-technologies-reduce-animal-use-in-research/

[13] FDA. FDA releases draft guidance on reducing testing on non-human primates for monoclonal antibodies. FDA. 2 Dec 2025. [accessed 5 Dec 2025] https://www.fda.gov/news-events/press-announcements/fda-releases-draft-guidance-reducing-testing-non-human-primates-monoclonal-antibodies

[14] Musah S, Bhattacharya R, Himmelfarb J. Kidney disease modeling with organoids and organs-on-chips. Annu Rev Biomed Eng. 2024;26(1):383–414. 38424088 10.1146/annurev-bioeng-072623-044010 PMC11479997

[15] Ingber DE. Human organs-on-chips for disease modelling, drug development and personalized medicine. Nat Rev Genet. 2022;23(8):467–491. 35338360 10.1038/s41576-022-00466-9 PMC8951665

[16] Chen W-Y, Evangelista EA, Yang J, Kelly EJ, Yeung CK. Kidney organoid and microphysiological kidney chip models to accelerate drug development and reduce animal testing. Front Pharmacol. 2021;12: Article 695920. 34381363 10.3389/fphar.2021.695920 PMC8350564

[17] Jiang S, Su H. Cellular crosstalk of mesangial cells and tubular epithelial cells in diabetic kidney disease. Cell Commun Signal. 2023;21(1):288. 37845726 10.1186/s12964-023-01323-w PMC10577991

[18] Hu S, Hang X, Wei Y, Wang H, Zhang L, Zhao L. Crosstalk among podocytes, glomerular endothelial cells and mesangial cells in diabetic kidney disease: an updated review. Cell Commun Signal. 2024;22(1):136. 38374141 10.1186/s12964-024-01502-3 PMC10875896

[19] Bejoy J, Farry JM, Peek JL, Cabatu MC, Williams FM, Welch RC, Qian ES, Woodard LE. Podocytes derived from human induced pluripotent stem cells: characterization, comparison, and modeling of diabetic kidney disease. Stem Cell Res Ther. 2022;13(1):355. 35883199 10.1186/s13287-022-03040-6 PMC9327311

[20] Xu P, Li K, Liu H, Xu A, Zhang Z, Yang Y, Lai X, Hao K, Fang K, Lai Z, et al. Inhibition of AMPKα pathway by podocyte GOLM1 exacerbates diabetic nephrology in mice. Adv Sci. 2025;12(37): Article e05695. 10.1002/advs.202505695 PMC12499477 40652516

[21] Pan X, Phanish MK, Baines DL, Dockrell ME. High glucose–induced Smad3 linker phosphorylation and CCN2 expression are inhibited by dapagliflozin in a diabetic tubule epithelial cell model. Bioscience Rep. 2021;41(6): Article BSR20203947. 10.1042/BSR20203947 PMC8220447 34003249

[22] Souren NY, Fusenig NE, Heck S, Dirks WG, Capes-Davis A, Bianchini F, Plass C. Cell line authentication: A necessity for reproducible biomedical research. EMBO J. 2022;41(14): Article e111307. 35758134 10.15252/embj.2022111307 PMC9289526

[23] Liu XQ, Jiang L, Li YY, Huang YB, Hu XR, Zhu W, Wang X, Wu YG, Meng XM, Qi XM. Wogonin protects glomerular podocytes by targeting Bcl-2-mediated autophagy and apoptosis in diabetic kidney disease. Acta Pharmacol Sin. 2022;43(1):96–110. 34253875 10.1038/s41401-021-00721-5 PMC8724322

[24] Pei X, Liu J, Wei Y, Zhu Y, Li Y, Wang Q, Zhuang L, Jin G. PINK1 affects mitochondrial oxidative phosphorylation by regulating MFN2 to alleviate diabetic kidney disease. Int Immunopharmacol. 2025;157: Article 114786. 40319746 10.1016/j.intimp.2025.114786

[25] Morizane R, Bonventre JV. Generation of nephron progenitor cells and kidney organoids from human pluripotent stem cells. Nat Protoc. 2017;12(1):195–207. 28005067 10.1038/nprot.2016.170 PMC5278902

[26] Barreto AD, Jiang B, Burt MA, Dimitrakakis N, Musah S. Discovery of kidney disease targets using multimodal human podocyte injury models. Stem Cell Rep. 2026;21(4): Article 102868. 10.1016/j.stemcr.2026.102868 PMC13083808 41932339

[27] Bejoy J, Qian ES, Woodard LE. Tissue culture models of AKI: From tubule cells to human kidney organoids. J Am Soc Nephrol. 2022;33(3):487–501. 35031569 10.1681/ASN.2021050693 PMC8975068

[28] Feitz WJC, van Setten PA, van der Velden TJAM, Licht C, van den Heuvel LPJW, van de Kar NCAJ. Cell biological responses after Shiga toxin-1 exposure to primary human glomerular microvascular endothelial cells from pediatric and adult origin. Int J Mol Sci. 2021;22(11):5615. 34070679 10.3390/ijms22115615 PMC8199108

[29] Lechner C, Mönning U, Reichel A, Fricker G. Potential and limits of kidney cells for evaluation of renal excretion. Pharmaceuticals. 2021;14(9):908. 34577608 10.3390/ph14090908 PMC8464824

[30] Esch EW, Bahinski A, Huh D. Organs-on-chips at the frontiers of drug discovery. Nat Rev Drug Discov. 2015;14(4):248–260. 25792263 10.1038/nrd4539 PMC4826389

[31] Nguyen VVT, Gkouzioti V, Maass C, Verhaar MC, Vernooij RWM, van Balkom BWM. A systematic review of kidney-on-a-chip-based models to study human renal (patho-)physiology. Dis Model Mech. 2023;16(6): Article dmm050113. 37334839 10.1242/dmm.050113 PMC10309579

[32] Buzhor E, Harari-Steinberg O, Omer D, Metsuyanim S, Jacob-Hirsch J, Noiman T, Dotan Z, Goldstein RS, Dekel B. Kidney spheroids recapitulate tubular organoids leading to enhanced tubulogenic potency of human kidney-derived cells. Tissue Eng Part A. 2011;17(17–18):2305–2319. 21542667 10.1089/ten.TEA.2010.0595

[33] Abe H, Sakurai A, Ochi A. Induction of steady-state glomeruloid sphere by self-assembly from human embryonic kidney cells. Biochem Biophys Res Commun. 2019;508(2):654–659. 30522864 10.1016/j.bbrc.2018.11.160

[34] Kang HM, Lim JH, Noh KH, Park D, Cho H-S, Susztak K, Jung C-R. Effective reconstruction of functional organotypic kidney spheroid for *in vitro* nephrotoxicity studies. Sci Rep. 2019;9(1):17610. 31772214 10.1038/s41598-019-53855-2 PMC6879515

[35] Giannuzzi F, Picerno A, Maiullari S, Montenegro F, Cicirelli A, Stasi A, De Palma G, Di Lorenzo VF, Pertosa GB, Pontrelli P, et al. Unveiling spontaneous renal tubule-like structures from human adult renal progenitor cell spheroids derived from urine. Stem Cells Transl Med. 2025;14(3): Article szaf002. 40156847 10.1093/stcltm/szaf002 PMC11954590

[36] Palau V, Nugraha B, Emmert M, Hoerstrup S, Pascual Santos J, Soler Romeo MJ, Riera M. Human kidney proximal tubular 3D spheroids to study the role of ADAM17 in diabetic nephropathy. Nephrol Dial Transplant. 2020; 35(Supplement_3): Article gfaa139.SO026.

[37] Tuffin J, Chesor M, Kuzmuk V, Johnson T, Satchell SC, Welsh GI, Saleem MA. GlomSpheres as a 3D co-culture spheroid model of the kidney glomerulus for rapid drug-screening. Commun Biol. 2021;4(1):1351. 34857869 10.1038/s42003-021-02868-7 PMC8640035

[38] Nayak P, Bentivoglio V, Varani M, Signore A. Three-dimensional in vitro tumor spheroid models for evaluation of anticancer therapy: Recent updates. Cancers. 2023;15(19):4846. 37835541 10.3390/cancers15194846 PMC10571930

[39] Huch M, Knoblich JA, Lutolf MP, Martinez-Arias A. The hope and the hype of organoid research. Development. 2017;144(6):938–941. 28292837 10.1242/dev.150201

[40] Sutherland TE, Dyer DP, Allen JE. The extracellular matrix and the immune system: A mutually dependent relationship. Science. 2023;379(6633): Article eabp8964. 36795835 10.1126/science.abp8964

[41] Li C, An N, Song Q, Hu Y, Yin W, Wang Q, Le Y, Pan W, Yan X, Wang Y, et al. Enhancing organoid culture: Harnessing the potential of decellularized extracellular matrix hydrogels for mimicking microenvironments. J Biomed Sci. 2024;31(1):96. 39334251 10.1186/s12929-024-01086-7 PMC11429032

[42] Bülow RD, Boor P. Extracellular matrix in kidney fibrosis: More than just a scaffold. J Histochem Cytochem. 2019;67(9):643–661. 31116062 10.1369/0022155419849388 PMC6713975

[43] Garreta E, Moya-Rull D, Marco A, Amato G, Ullate-Agote A, Tarantino C, Gallo M, Esporrín-Ubieto D, Centeno A, Vilas-Zornoza A, et al. Natural hydrogels support kidney organoid generation and promote in vitro angiogenesis. Adv Mater. 2024;36(34): Article e2400306. 38762768 10.1002/adma.202400306

[44] Clerkin S, Singh K, Davis JL, Treacy NJ, Krupa I, Reynaud EG, Lees RM, Needham SR, MacWhite-Begg D, Wychowaniec JK, et al. Tuneable gelatin methacryloyl (GelMA) hydrogels for the directed specification of renal cell types for hiPSC-derived kidney organoid maturation. Biomaterials. 2025;322: Article 123349. 40315627 10.1016/j.biomaterials.2025.123349

[45] Hu M, Azeloglu EU, Ron A, Tran-Ba K-H, Calizo RC, Tavassoly I, Bhattacharya S, Jayaraman G, Chen Y, Rabinovich V, et al. A biomimetic gelatin-based platform elicits a pro-differentiation effect on podocytes through mechanotransduction. Sci Rep. 2017;7(1):43934. 28262745 10.1038/srep43934 PMC5338254

[46] Treacy NJ, Clerkin S, Davis JL, Kennedy C, Miller AF, Saiani A, Wychowaniec JK, Brougham DF, Crean J. Growth and differentiation of human induced pluripotent stem cell (hiPSC)-derived kidney organoids using fully synthetic peptide hydrogels. Bioact Mater. 2022;21:142–156. 36093324 10.1016/j.bioactmat.2022.08.003 PMC9420433

[47] Guimarães CF, Gasperini L, Marques AP, Reis RL. The stiffness of living tissues and its implications for tissue engineering. Nat Rev Mater. 2020;5(5):351–370.

[48] Zhang C, Shen Y, Huang M, Wang G, Miao Q, Shi H, Gao R, Wang K, Luo M. Dynamic hydrogel mechanics in organoid engineering: From matrix design to translational paradigms. Bioact Mater. 2025;55:144–170. 41035429 10.1016/j.bioactmat.2025.09.021 PMC12481939

[49] Kumar SV, Er PX, Lawlor KT, Motazedian A, Scurr M, Ghobrial I, Combes AN, Zappia L, Oshlack A, Stanley EG, et al. Kidney micro-organoids in suspension culture as a scalable source of human pluripotent stem cell-derived kidney cells. Development. 2019;146(5): Article dev172361. 30846463 10.1242/dev.172361 PMC6432662

[50] Knight E, Przyborski S. Advances in 3D cell culture technologies enabling tissue-like structures to be created *in vitro*. J Anat. 2014;227(6):746–756. 25411113 10.1111/joa.12257 PMC4694114

[51] Tran T, Song CJ, Nguyen T, Cheng S-Y, McMahon JA, Yang R, Guo Q, Der B, Lindström NO, Lin DC-H, et al. A scalable organoid model of human autosomal dominant polycystic kidney disease for disease mechanism and drug discovery. Cell Stem Cell. 2022;29(7):1083–1101.e7. 35803227 10.1016/j.stem.2022.06.005 PMC11088748

[52] Przepiorski A, Sander V, Tran T, Hollywood JA, Sorrenson B, Shih J-H, Wolvetang EJ, McMahon AP, Holm TM, Davidson AJ. A simple bioreactor-based method to generate kidney organoids from pluripotent stem cells. Stem Cell Rep. 2018;11(2):470–484. 10.1016/j.stemcr.2018.06.018 PMC6092837 30033089

[53] Kim D, Lim H, Youn J, Park TE, Kim DS. Scalable production of uniform and mature organoids in a 3D geometrically-engineered permeable membrane. Nat Commun. 2024;15(1):9420. 39482314 10.1038/s41467-024-53073-z PMC11528013

[54] Hu Y, Zhu T, Cui H, Cui H. Integrating 3D bioprinting and organoids to better recapitulate the complexity of cellular microenvironments for tissue engineering. Adv Healthc Mater. 2025;14(3): Article e2403762. 39648636 10.1002/adhm.202403762

[55] Maharjan S, Ma C, Singh B, Kang H, Orive G, Yao J, Zhang YS. Advanced 3D imaging and organoid bioprinting for biomedical research and therapeutic applications. Adv Drug Deliv Rev. 2024;208: Article 115237. 38447931 10.1016/j.addr.2024.115237 PMC11031334

[56] Shin J, Tabatabaei Rezaei N, Choi S, Li Z, Kim DH, Kim K. Photocrosslinkable kidney decellularized extracellular matrix-based bioink for 3D bioprinting. Adv Healthc Mater. 2025;14(24): Article e2501616. 40665936 10.1002/adhm.202501616 PMC12447028

[57] Perin F, Ricci A, Fagiolino S, Rak-Raszewska A, Kearney H, Ramis J, Bereen I, Baker M, Maniglio D, Motta A, et al. Bioprinting of Alginate-Norbornene bioinks to create a versatile platform for kidney *in vitro* modeling. Bioact Mater. 2025;49:550–563. 40212782 10.1016/j.bioactmat.2025.03.010 PMC11982478

[58] Formica C, Addario G, Fagiolino S, Moroni L, Mota C. Microfluidic bioprinting as a tool to produce hiPSCs-derived renal organoids. Biofabrication. 2025;17(3): Article 035016. 10.1088/1758-5090/addb7e 40398439

[59] Konoe R, Morizane R. Strategies for improving vascularization in kidney organoids: A review of current trends. Biology. 2023;12(4):503. 37106704 10.3390/biology12040503 PMC10135596

[60] Koning M, Dumas SJ, Avramut MC, Koning RI, Meta E, Lievers E, Wiersma LE, Borri M, Liang X, Xie L, et al. Vasculogenesis in kidney organoids upon transplantation. NPJ Regen Med. 2022;7(1):40. 35986027 10.1038/s41536-022-00237-4 PMC9391397

[61] Jansen J, van den Berge BT, van den Broek M, Maas RJ, Daviran D, Willemsen B, Roverts R, van der Kruit M, Kuppe C, Reimer KC, et al. Human pluripotent stem cell-derived kidney organoids for personalized congenital and idiopathic nephrotic syndrome modeling. Development. 2022;149(9): Article dev200198. 35417019 10.1242/dev.200198 PMC9148570

[62] Gaykema LH, van Nieuwland RY, Lievers E, Moerkerk WB, de Klerk JA, Dumas SJ, Kers J, Zaldumbide A, van den Berg CW, Rabelink TJ. T-cell mediated immune rejection of beta-2-microglobulin knockout induced pluripotent stem cell-derived kidney organoids. Stem Cells Transl Med. 2023;13(1):69–82 10.1093/stcltm/szad069 PMC10785221 37843402

[63] Bhatia SN, Ingber DE. Microfluidic organs-on-chips. Nat Biotechnol. 2014;32(8):760–772. 25093883 10.1038/nbt.2989

[64] Huang W, Chen Y-Y, He F-F, Zhang C. Revolutionizing nephrology research: Expanding horizons with kidney-on-a-chip and beyond. Front Bioeng Biotechnol. 2024;12:1373386. 38605984 10.3389/fbioe.2024.1373386 PMC11007038

[65] Dong R, Xu Y. Glomerular cell cross talk in diabetic kidney diseases. J Diabetes. 2022;14(8):514–523. 35999686 10.1111/1753-0407.13304 PMC9426281

[66] Li X, Zhang Y, Xing X, Li M, Liu Y, Ajing X, Zhang J. Podocyte injury of diabetic nephropathy: Novel mechanism discovery and therapeutic prospects. Biomed Pharmacother. 2023;168: Article 115670. 37837883 10.1016/j.biopha.2023.115670

[67] Petrosyan A, Cravedi P, Villani V, Angeletti A, Manrique J, Renieri A, De Filippo RE, Perin L, Da Sacco S. A glomerulus-on-a-chip to recapitulate the human glomerular filtration barrier. Nat Commun. 2019;10(1):3656. 31409793 10.1038/s41467-019-11577-z PMC6692336

[68] Mou X, Shah J, Roye Y, Du C, Musah S. An ultrathin membrane mediates tissue-specific morphogenesis and barrier function in a human kidney chip. Sci Adv. 2024;10(23): Article eadn2689. 38838141 10.1126/sciadv.adn2689 PMC11152122

[69] Mussoni C, Rederer A, Stepanenko V, Würthner F, Stahlhut P, Groll J, Schiffer M, Ahmad T, Müller-Deile J. Biofabrication of a filtration barrier by integrating electrospun membranes and flow in a glomerular co-culture. Adv Healthc Mater. 2025;14(23):2501235. 40605638 10.1002/adhm.202501235 PMC12417763

[70] Kim SY, Choi YY, Kwon EJ, Seo S, Kim WY, Park SH, Park S, Chin HJ, Na KY, Kim S. Characterizing glomerular barrier dysfunction with patient-derived serum in glomerulus-on-a-chip models: Unveiling new insights into glomerulonephritis. Int J Mol Sci. 2024;25(10):5121. 38791159 10.3390/ijms25105121 PMC11121116

[71] Beckmann SB, Salib M, Fenton RA, Hoorn EJ. Toward assessment of tubular function in kidney disease. J Am Soc Nephrol. 2025;36(6):1201–1203. 40111418 10.1681/ASN.0000000705 PMC12147954

[72] Jing B, Yan L, Li J, Luo P, Ai X, Tu P. Functional evaluation and nephrotoxicity assessment of human renal proximal tubule cells on a chip. Biosensors. 2022;12(9):718. 36140103 10.3390/bios12090718 PMC9496563

[73] Rayner SG, Phong KT, Xue J, Lih D, Shankland SJ, Kelly EJ, Himmelfarb J, Zheng Y. Reconstructing the human renal vascular-tubular unit in vitro. Adv Healthc Mater. 2018;7(23): Article e1801120. 30379416 10.1002/adhm.201801120 PMC6478624

[74] Chapron A, Chapron BD, Hailey DW, Chang SY, Imaoka T, Thummel KE, Kelly E, Himmelfarb J, Shen D, Yeung CK. An improved vascularized, dual-channel microphysiological system facilitates modeling of proximal tubular solute secretion. ACS Pharmacol Transl Sci. 2020;3(3):496–508. 32566915 10.1021/acsptsci.9b00078 PMC7296546

[75] Li Y, Sun K, Shao Y, Wang C, Xue F, Chu C, Gu Z, Chen Z, Bai J. Next-generation approaches for biomedical materials evaluation: Microfluidics and organ-on-a-chip technologies. Adv Healthc Mater. 2025;14(1): Article e2402611. 39440635 10.1002/adhm.202402611

[76] Lin NYC, Homan KA, Robinson SS, Kolesky DB, Duarte N, Moisan A, Lewis JA. Renal reabsorption in 3D vascularized proximal tubule models. Proc Natl Acad Sci USA. 2019;116(12):5399–5404. 30833403 10.1073/pnas.1815208116 PMC6431199

[77] Guimaraes APP, Calori IR, Stilhano RS, Tedesco AC. Renal proximal tubule-on-a-chip in PDMS: Fabrication, functionalization, and RPTEC:HUVEC co-culture evaluation. Biofabrication. 2024;16(2): Article 025024. 10.1088/1758-5090/ad2d2f 38408383

[78] Nishimura Y. Revolutionizing renal research: The future of kidney-on-a-chip in biotechnology. Regen Ther. 2024;26:275–280. 38993536 10.1016/j.reth.2024.06.006 PMC11237358

[79] Liu A, Wang X, Hu X, Deng Y, Wen X, Lin B, Zhou M, Wang W, Luo Y, Deng J, et al. Core fucosylation involvement in the paracrine regulation of proteinuria-induced renal interstitial fibrosis evaluated with the use of a microfluidic chip. Acta Biomater. 2022;142:99–112. 35189379 10.1016/j.actbio.2022.02.020

[80] Bouwens D, Kabgani N, Bergerbit C, Kim H, Ziegler S, Ijaz S, Abdallah A, Haraszti T, Maryam S, Omidinia-Anarkoli A, et al. A bioprinted and scalable model of human tubulo-interstitial kidney fibrosis. Biomaterials. 2025;316: Article 123009. 39705928 10.1016/j.biomaterials.2024.123009

[81] Addario G, Fernández-Pérez J, Formica C, Karyniotakis K, Herkens L, Djudjaj S, Boor P, Moroni L, Mota C. 3D humanized bioprinted tubulointerstitium model to emulate renal fibrosis in vitro. Adv Healthc Mater. 2024;13(29): Article e2400807. 39152919 10.1002/adhm.202400807 PMC11582511

[82] Theodorou C, Leatherby R, Dhanda R. Function of the nephron and the formation of urine. Anaesth Intensive Care Med. 2021;22(7):434–438.

[83] Qu Y, An F, Luo Y, Lu Y, Liu T, Zhao W, Lin B. A nephron model for study of drug-induced acute kidney injury and assessment of drug-induced nephrotoxicity. Biomaterials. 2018;155:41–53. 29169037 10.1016/j.biomaterials.2017.11.010

[84] Zhang SY, Mahler GJ. A glomerulus and proximal tubule microphysiological system simulating renal filtration, reabsorption, secretion, and toxicity. Lab Chip. 2023;23(2):272–284. 36514972 10.1039/d2lc00887d

[85] Bas-Cristóbal Menéndez A, Du Z, van den Bosch TPP, Othman A, Gaio N, Silvestri C, Quirós W, Lin H, Korevaar S, Merino A, et al. Creating a kidney organoid-vasculature interaction model using a novel organ-on-chip system. Sci Rep. 2022;12(1):20699. 36450835 10.1038/s41598-022-24945-5 PMC9712653

[86] Kroll KT, Homan KA, Uzel SG, Mata MM, Wolf KJ, Rubins JE, Lewis JA. A perfusable, vascularized kidney organoid-on-chip model. Biofabrication. 2024;16(4): Article 045003. 10.1088/1758-5090/ad5ac0 38906132

[87] Kiranmai G, Chameettachal S, Sriya Y, Duin S, Lode A, Gelinsky M, Akkineni AR, Pati F. Recent trends in the development of *in vitro* 3D kidney models. Biofabrication. 2025;17(2): Article 022010. 10.1088/1758-5090/adb999 39993331

[88] Phipson B, Er PX, Combes AN, Forbes TA, Howden SE, Zappia L, Yen H-J, Lawlor KT, Hale LJ, Sun J, et al. Evaluation of variability in human kidney organoids. Nat Methods. 2019;16(1):79–87. 30573816 10.1038/s41592-018-0253-2 PMC6634992

[89] Slyne J, Slattery C, McMorrow T, Ryan MP. New developments concerning the proximal tubule in diabetic nephropathy: *In vitro* models and mechanisms. Nephrol Dial Transplant. 2015;30(Suppl_4):iv60-7. 26209740 10.1093/ndt/gfv264

[90] Feng Q, Yu X, Xie J, Liu F, Zhang X, Li S, Wang Y, Pan S, Liu D, Liu Z. Phillygenin improves diabetic nephropathy by inhibiting inflammation and apoptosis via regulating TLR4/MyD88/NF-κB and PI3K/AKT/GSK3β signaling pathways. Phytomedicine. 2025;136: Article 156314. 39647467 10.1016/j.phymed.2024.156314

[91] Garreta E, Prado P, Stanifer ML, Monteil V, Marco A, Ullate-Agote A, Moya-Rull D, Vilas-Zornoza A, Tarantino C, Romero JP, et al. A diabetic milieu increases ACE2 expression and cellular susceptibility to SARS-CoV-2 infections in human kidney organoids and patient cells. Cell Metab. 2022;34(6):857–873.e9. 35561674 10.1016/j.cmet.2022.04.009 PMC9097013

[92] Spennati G, Regier MC, Ward HH, Das V, Karihaloo A, Freedman BS. Elevated glucose in kidney organoids induces tissue-intrinsic inflammation driving epithelial detachment. bioRxiv. 2025. 10.1101/2025.09.05.66941642753732

[93] Wimmer RA, Leopoldi A, Aichinger M, Wick N, Hantusch B, Novatchkova M, Taubenschmid J, Hämmerle M, Esk C, Bagley JA, et al. Human blood vessel organoids as a model of diabetic vasculopathy. Nature. 2019;565(7740):505–510. 30651639 10.1038/s41586-018-0858-8 PMC7116578

[94] Aceves JO, Heja S, Kobayashi K, Robinson SS, Miyoshi T, Matsumoto T, Schäffers OJ, Morizane R, Lewis JA. 3D proximal tubule-on-chip model derived from kidney organoids with improved drug uptake. Sci Rep. 2022;12(1):14997. 36056134 10.1038/s41598-022-19293-3 PMC9440090

[95] Wang L, Tao T, Su W, Yu H, Yu Y, Qin J. A disease model of diabetic nephropathy in a glomerulus-on-a-chip microdevice. Lab Chip. 2017;17(10):1749–1760. 28418422 10.1039/c7lc00134g

[96] Hall AM, Polesel M, Berquez M. The proximal tubule, protein uptake, and the riddle of the segments. Kidney Int. 2021;99(4):803–805. 33745544 10.1016/j.kint.2020.12.031

[97] Sung JH. Multi-organ-on-a-chip for pharmacokinetics and toxicokinetic study of drugs. Expert Opin Drug Metab Toxicol. 2021;17(8):969–986. 33764248 10.1080/17425255.2021.1908996

[98] Ronaldson-Bouchard K, Teles D, Yeager K, Tavakol DN, Zhao Y, Chramiec A, Tagore S, Summers M, Stylianos S, Tamargo M, et al. A multi-organ chip with matured tissue niches linked by vascular flow. Nat Biomed Eng. 2022;6(4):351–371. 35478225 10.1038/s41551-022-00882-6 PMC9250010

[99] Picollet-D’hahan N, Zuchowska A, Lemeunier I, Le Gac S. Multiorgan-on-a-chip: A systemic approach to model and decipher inter-organ communication. Trends Biotechnol. 2021;39(8):788–810. 33541718 10.1016/j.tibtech.2020.11.014

[100] Chang SY, Weber EJ, Sidorenko VS, Chapron A, Yeung CK, Gao C, Mao Q, Shen D, Wang J, Rosenquist TA, et al. Human liver-kidney model elucidates the mechanisms of aristolochic acid nephrotoxicity. JCI Insight. 2017;2(22): Article e95978. 29202460 10.1172/jci.insight.95978 PMC5752374

[101] Nguyen VVT, Ye S, Gkouzioti V, van Wolferen ME, Yengej FY, Melkert D, Siti S, de Jong B, Besseling PJ, Spee B, et al. A human kidney and liver organoid-based multi-organ-on-a-chip model to study the therapeutic effects and biodistribution of mesenchymal stromal cell-derived extracellular vesicles. J Extracell Vesicles. 2022;11(11): Article e12280. 36382606 10.1002/jev2.12280 PMC9667402

[102] Juguilon C, Khosravi R, Radisic M, Wu JC. In vitro modeling of interorgan crosstalk: Multi-organ-on-a-chip for studying cardiovascular-kidney-metabolic syndrome. Circ Res. 2025;136(11):1476–1493. 40403116 10.1161/CIRCRESAHA.125.325497 PMC12180411

[103] Giordano L, Mihaila SM, Amirabadi HE, Masereeuw R. Microphysiological systems to recapitulate the gut–kidney axis. Trends Biotechnol. 2021;39(8):811–823. 33419585 10.1016/j.tibtech.2020.12.001

[104] Li Z, Jiang L, Zhu Y, Su W, Xu C, Tao T, Shi Y, Qin J. Assessment of hepatic metabolism-dependent nephrotoxicity on an organs-on-a-chip microdevice. Toxicol In Vitro. 2018;46:1–8. 28986290 10.1016/j.tiv.2017.10.005

[105] Kim JJ, Park JY, Nguyen VV-T, Bae M, Kim M, Jang J, Won JY, Cho D-W. Pathophysiological reconstruction of a tissue-specific multiple-organ on-a-chip for type 2 diabetes emulation using 3D cell printing. Adv Funct Mater. 2023;33(22):2213649.

[106] Duan X, Zhang X, Chen J, Xiao M, Zhao W, Liu S, Sui G. Association of PM_2.5_ with insulin resistance signaling pathways on a microfluidic liver-kidney microphysiological system (LK-MPS) device. Anal Chem. 2021;93(28):9835–9844. 34232631 10.1021/acs.analchem.1c01384

[107] Gabbin B, Meraviglia V, Angenent ML, Ward-van Oostwaard D, Sol W, Mummery CL, Rabelink TJ, van Meer BJ, van den Berg CW, Bellin M. Heart and kidney organoids maintain organ-specific function in a microfluidic system. Mater Today Bio. 2023;23: Article 100818. 10.1016/j.mtbio.2023.100818 PMC10550812 37810749

[108] Herland A, Maoz BM, Das D, Somayaji MR, Prantil-Baun R, Novak R, Cronce M, Huffstater T, Jeanty SSF, Ingram M, et al. Quantitative prediction of human drug pharmacokinetic responses using multiple vascularized organ chips coupled by fluid transfer. Nat Biomed Eng. 2020;4(4):421–436. 31988459 10.1038/s41551-019-0498-9 PMC8011576

[109] Novak R, Ingram M, Marquez S, Das D, Delahanty A, Herland A, Maoz BM, Jeanty SS, Somayaji MR, Burt M, et al. Robotic fluidic coupling and interrogation of multiple vascularized organ chips. Nat Biomed Eng. 2020;4(4):407–420. 31988458 10.1038/s41551-019-0497-x PMC8057865

[110] Wu H, Humphreys BD. Single cell sequencing and kidney organoids generated from pluripotent stem cells. Clin J Am Soc Nephrol. 2020;15(4):550–556. 31992574 10.2215/CJN.07470619 PMC7133134

[111] Chen D, Shao M, Song Y, Ren G, Guo F, Fan X, Wang Y, Zhang W, Qin G. Single-cell RNA-seq with spatial transcriptomics to create an atlas of human diabetic kidney disease. FASEB J. 2023;37(6): Article e22938. 37130011 10.1096/fj.202202013RR

[112] Wilson SB, Howden SE, Vanslambrouck JM, Dorison A, Alquicira-Hernandez J, Powell JE, Little MH. DevKidCC allows for robust classification and direct comparisons of kidney organoid datasets. Genome Med. 2022;14(1):19. 35189942 10.1186/s13073-022-01023-z PMC8862535

[113] Pacesa M, Pelea O, Jinek M. Past, present, and future of CRISPR genome editing technologies. Cell. 2024;187(5):1076–1100. 38428389 10.1016/j.cell.2024.01.042

[114] Liu E, Radmanesh B, Chung BH, Donnan MD, Yi D, Dadi A, Smith KD, Himmelfarb J, Li M, Freedman BS, et al. Profiling *APOL1* nephropathy risk variants in genome-edited kidney organoids with single-cell transcriptomics. Kidney360. 2020;1(3):203–215. 32656538 10.34067/KID.0000422019 PMC7351353

[115] D’Ambrosio V, Huimei C, Vo N, Siew K, Evans RD, Freedman B, Pesce F. CRISPR and gene editing for kidney diseases: Where are we? Clin Kidney J. 2025;18(9): Article sfaf246. 40927382 10.1093/ckj/sfaf246 PMC12415518

[116] Bai L, Wu Y, Li G, Zhang W, Zhang H, Su J. AI-enabled organoids: Construction, analysis, and application. Bioact Mater. 2024;31:525–548. 37746662 10.1016/j.bioactmat.2023.09.005 PMC10511344

[117] Tan S, Ding Y, Wang W, Rao J, Cheng F, Zhang Q, Xu T, Hu T, Hu Q, Ye Z, et al. Development of an AI model for DILI-level prediction using liver organoid brightfield images. Commun Biol. 2025;8(1):886. 40483291 10.1038/s42003-025-08205-6 PMC12145446

[118] Gijzen L, Bokkers M, Hanamsagar R, Olivier T, Burton TP, Tool LM, Rahman MF, Lowman J, Savova V, Means TK, et al. An immunocompetent human kidney on-a-chip model to study renal inflammation and immune-mediated injury. Biofabrication. 2024;17(1): Article 015040. 10.1088/1758-5090/ad9fdf 39681083

[119] Kroll KT, Mata MM, Homan KA, Micallef V, Carpy A, Hiratsuka K, Morizane R, Moisan A, Gubler M, Walz AC, et al. Immune-infiltrated kidney organoid-on-chip model for assessing T cell bispecific antibodies. Proc Natl Acad Sci USA. 2023;120(35): Article e2305322120. 37603766 10.1073/pnas.2305322120 PMC10467620

[120] Chen Z, Huang J, Zhang J, Xu Z, Li Q, Ouyang J, Yan Y, Sun S, Ye H, Wang F, et al. A storm in a teacup—A biomimetic lung microphysiological system in conjunction with a deep-learning algorithm to monitor lung pathological and inflammatory reactions. Biosens Bioelectron. 2023;219: Article 114772. 36272347 10.1016/j.bios.2022.114772

[121] Ramadan Q, Zourob M. Organ-on-a-chip engineering: Toward bridging the gap between lab and industry. Biomicrofluidics. 2020;14(4): Article 041501. 32699563 10.1063/5.0011583 PMC7367691

[122] Ching T, Toh Y-C, Hashimoto M, Zhang YS. Bridging the academia-to-industry gap: Organ-on-a-chip platforms for safety and toxicology assessment. Trends Pharmacol Sci. 2021;42(9):715–728. 34187693 10.1016/j.tips.2021.05.007 PMC8364498

[123] Bae E, Yu M-Y, Moon J-J, Kim J-E, Lee S, Han S-W, Park D-J, Kim Y-S, Yang S-H. Renoprotective effect of KLF2 on glomerular endothelial dysfunction in hypertensive nephropathy. Cells. 2022;11(5):762. 35269384 10.3390/cells11050762 PMC8909753

[124] Bajaj P, Chung G, Pye K, Yukawa T, Imanishi A, Takai Y, Brown C, Wagoner MP. Freshly isolated primary human proximal tubule cells as an in vitro model for the detection of renal tubular toxicity. Toxicology. 2020;442: Article 152535. 32622972 10.1016/j.tox.2020.152535

[125] Guo C, Wang Y, Piao Y, Rao X, Yin D. Chrysophanol inhibits the progression of diabetic nephropathy via inactivation of TGF-β pathway. Drug Des Devel Ther. 2020;14:4951–4962. 10.2147/DDDT.S274191 PMC7678702 33235436

[126] Wu L, Li L, Wang X, Wu H, Li M, Wang Y, Sheng P, An X, Yan M. The inhibition of rutin on Src kinase blocks high glucose-induced EGFR/ERK transactivation in diabetic nephropathy by integrative approach of network pharmacology and experimental verification. Phytomedicine. 2024;135: Article 156220. 39541664 10.1016/j.phymed.2024.156220

[127] Wu J, Wang Z, Cai M, Wang X, Lo B, Li Q, He JC, Lee K, Fu J. GPR56 promotes diabetic kidney disease through eNOS regulation in glomerular endothelial cells. Diabetes. 2023;72(11):1652–1663. 37579299 10.2337/db23-0124 PMC10588296

[128] Xu Z, Jia K, Wang H, Gao F, Zhao S, Li F, Hao J. METTL14-regulated PI3K/Akt signaling pathway via PTEN affects HDAC5-mediated epithelial–mesenchymal transition of renal tubular cells in diabetic kidney disease. Cell Death Dis. 2021;12(1):32. 33414476 10.1038/s41419-020-03312-0 PMC7791055

[129] Phung HM, Lee S, Hwang JH, Kang KS. Preventive effect of muscone against cisplatin nephrotoxicity in LLC-PK1 cells. Biomolecules. 2020;10(10):1444. 33076219 10.3390/biom10101444 PMC7602442

[130] Mboni-Johnston IM, Kouidrat NMZ, Hirsch C, Weber AG, Meißner A, Adjaye J, Schupp N. Sensitivity of human induced pluripotent stem cells and thereof differentiated kidney proximal tubular cells towards selected nephrotoxins. Int J Mol Sci. 2023;25(1):81. 38203251 10.3390/ijms25010081 PMC10779191

[131] Zhang SY, Mahler GJ. Modelling renal filtration and reabsorption processes in a human glomerulus and proximal tubule microphysiological system. Micromachines. 2021;12(8):983. 34442605 10.3390/mi12080983 PMC8398588

[132] Vidal Yucha SE, Quackenbush D, Chu T, Lo F, Sutherland JJ, Kuzu G, Roberts C, Luna F, Barnes SW, Walker J, et al. 3D, human renal proximal tubule (RPTEC-TERT1) organoids ‘tubuloids’ for translatable evaluation of nephrotoxins in high-throughput. PLoS One. 2022;17(11): Article e0277937. 36409750 10.1371/journal.pone.0277937 PMC9678317

[133] Kim JW, Nam SA, Yi J, Kim JY, Lee JY, Park SY, Sen T, Choi YM, Lee JY, Kim HL, et al. Kidney decellularized extracellular matrix enhanced the vascularization and maturation of human kidney organoids. Adv Sci. 2022;9(15): Article e2103526. 10.1002/advs.202103526 PMC9130892 35322595

